# Advanced Numerical Modeling of Powder Bed Fusion: From Physics-Based Simulations to AI-Augmented Digital Twins

**DOI:** 10.3390/ma19020426

**Published:** 2026-01-21

**Authors:** Łukasz Łach, Dmytro Svyetlichnyy

**Affiliations:** AGH University of Krakow, Faculty of Metals Engineering and Industrial Computer Science, al. Mickiewicza 30, 30-059 Krakow, Poland; lach@agh.edu.pl

**Keywords:** Powder Bed Fusion (PBF), additive manufacturing, multiscale modeling, machine learning, digital twins, process monitoring, defect prediction, multiphysics simulation, industrial applications

## Abstract

Powder bed fusion (PBF) is a widely adopted additive manufacturing (AM) process category that enables high-resolution fabrication across metals, polymers, ceramics, and composites. However, its inherent process complexity demands robust modeling to ensure quality, reliability, and scalability. This review provides a critical synthesis of advances in physics-based simulations, machine learning, and digital twin frameworks for PBF. We analyze progress across scales—from micro-scale melt pool dynamics and mesoscale track stability to part-scale residual stress predictions—while highlighting the growing role of hybrid physics–data-driven approaches in capturing process–structure–property (PSP) relationships. Special emphasis is given to the integration of real-time sensing, multi-scale modeling, and AI-enhanced optimization, which together form the foundation of emerging PBF digital twins. Key challenges—including computational cost, data scarcity, and model interoperability—are critically examined, alongside opportunities for scalable, interpretable, and industry-ready digital twin platforms. By outlining both the current state-of-the-art and future research priorities, this review positions digital twins as a transformative paradigm for advancing PBF toward reliable, high-quality, and industrially scalable manufacturing.

## 1. Introduction

Additive manufacturing (AM) has transitioned over the past three decades from a rapid prototyping tool to a disruptive manufacturing technology capable of producing functional, high-performance components across aerospace, biomedical, automotive, and energy sectors [1]. Unlike subtractive processes, AM builds parts layer by layer directly from digital models, enabling unprecedented design freedom, near-net-shape fabrication, and efficient material utilization. Within the family of AM processes, powder bed fusion (PBF) has emerged as one of the most advanced and industrially relevant methods for producing metallic components with exceptional geometric precision and tailored microstructures [2].

PBF encompasses laser-based processes such as selective laser melting (SLM) and selective laser sintering (SLS), grouped recently in the laser beam PBF (PBF-LB or LPBF) or electron beam-based methods like electron beam melting (EBM or PBF-EB). In these processes, thin layers of metallic powder are selectively melted or sintered using a focused energy source under a controlled atmosphere or vacuum. The layer-by-layer consolidation enables the fabrication of complex geometries—including lattice structures, internal channels, and graded materials—that are often impossible to achieve through conventional methods. Furthermore, PBF is increasingly used for processing advanced materials such as titanium alloys (Ti-6Al-4V), stainless steels (316L), nickel-based superalloys (Inconel 718, Hastelloy X), and high-entropy alloys, where microstructural control directly influences the final part’s mechanical and functional performance [3].

Despite these advantages, PBF remains a complex, highly nonlinear process governed by the interplay of multi-scale and multiphysics phenomena. Localized energy input results in steep thermal gradients, rapid solidification, and transient melt pool dynamics, while layer-wise powder deposition introduces stochastic variability in packing density and morphology. These factors can lead to undesirable defects, such as porosity, lack of fusion, keyholing, cracking, and anisotropic mechanical properties [4]. The strong coupling between process parameters, transient thermal history, microstructural evolution, and residual stress development makes process optimization challenging [1].

Modeling and simulation have become indispensable for understanding, predicting, and controlling these complex interactions. Early modeling efforts—based on empirical correlations and analytical heat transfer equations—provided valuable but limited insights, often applicable only under narrow process conditions. As computational capabilities advanced, physics-based numerical models emerged, offering deeper process fidelity. Techniques such as the finite element method (FEM) have been used for thermal and mechanical analysis, computational fluid dynamics (CFD) for melt pool flow and keyhole formation, discrete element method (DEM) for powder spreading and packing behavior, and phase-field and cellular automata (CA) models for predicting microstructure and texture formation during solidification [2]. These methods have been instrumental in elucidating the process–structure–property (PSP) relationships in PBF, enabling the virtual exploration of parameter space and targeted optimization of part performance.

However, even the most advanced physics-based simulations face significant practical limitations. They are computationally expensive, often requiring high-performance computing resources, and may be difficult to generalize across materials, machines, and building geometries. Furthermore, their predictive power is limited by uncertainties in material property data at extreme processing conditions and by the lack of integration with real-time manufacturing data [5].

The emergence of digital twins—dynamic virtual replicas integrating simulation, in situ monitoring, and machine learning (detailed in Section 6)—represents a paradigm shift toward real-time process control [6]. Unlike static models, digital twins enable adaptive parameter adjustment during fabrication, bridging the gap between prediction and manufacturing to achieve "first-time-right" production and accelerate qualification cycles [7]. This integration of modeling, sensing, and control unlocks new capabilities for microstructure tailoring and functional optimization of the final product [6].

This review traces the evolution of PBF modeling from classical physics-based simulations to AI-augmented digital twins, emphasizing how multi-scale frameworks, hybrid approaches, and sensor fusion are reshaping the understanding of process–structure–property. By systematically evaluating advances, limitations, and open challenges, we establish a roadmap for scalable, interpretable digital twins capable of driving high-quality PBF manufacturing and accelerating integration into next-generation additive manufacturing.

### 1.1. Novelty and Significance

The modeling of powder bed fusion (PBF) processes has been the subject of numerous recent reviews, each addressing specific aspects of the field but leaving critical gaps in integration and scope. Bayat et al. [8] provided comprehensive coverage of multi-scale and multiphysics simulations emphasizing finite element methods and thermal–mechanical coupling, yet offered limited treatment of data-driven approaches and digital twin integration. Zinovieva et al. [9] focused specifically on microstructure-informed mechanical modeling with detailed crystal plasticity frameworks, but did not encompass the complete process chain from powder spreading through solidification or address optimization and digital twin architectures. Chowdhury et al. [10] and Sarkar et al. [2] delivered extensive surveys of numerical techniques, including finite element, computational fluid dynamics, and discrete element methods, though both preceded the recent surge in hybrid physics–machine learning approaches and industrial digital twin implementations. In the digital twin domain, Ahsan et al. [11] and Ben Amor et al. [12] cataloged applications and architectural frameworks across additive manufacturing, but lacked deep integration with underlying physics-based modeling foundations and specific optimization strategies essential for PBF deployment. This review addresses these gaps by synthesizing the complete PBF modeling ecosystem within an integrated, process-chain-oriented framework that systematically connects powder spreading, laser powder interaction, melt pool dynamics, solidification, thermomechanical response, and defect formation. The work establishes explicit cross linkages between physics-based simulations, in situ monitoring and sensor fusion, digital twin architectures, and optimization frameworks—revealing how advances in one domain enable progress in others. A multi-dimensional evaluation framework is introduced and applied uniformly across all modeling approaches, assessing physics fidelity, validation level, computational cost, real-time capability, transferability, and industrial readiness, thereby transforming the review from a descriptive catalog into a decision-support tool for method selection. A comprehensive treatment of emerging hybrid physics–machine learning methodologies, multi-sensor data fusion, and adaptive control strategies is provided with a critical assessment of current limitations and industrial deployment pathways. The review concludes with a time-bounded research roadmap distinguishing near-term priorities (validation benchmarks, open-source frameworks, GPU-accelerated simulations) from mid-term goals (industrial-scale digital twins, multi-material modeling, uncertainty quantification for certification), explicitly connecting research advances to industrial needs and providing actionable guidance for advancing PBF toward certifiable, production-ready manufacturing.

### 1.2. Review Method

A systematic literature review was conducted targeting peer-reviewed publications primarily from 2020 to 2025, with the selective inclusion of foundational published works before 2020 (8% of the corpus). Five databases were queried (Scopus, Web of Science, ScienceDirect, IEEE Xplore, SpringerLink) using Boolean search terms combining PBF process variants (“powder bed fusion” OR “LPBF” OR “L-PBF” OR “selective laser melting”) AND modeling approaches (“simulation” OR “machine learning” OR “digital twin” OR “physics-informed” OR “process monitoring”).

Inclusion criteria: Peer-reviewed journal articles, conference proceedings, and reviews in English focused on PBF physics-based modeling, data-driven/ML frameworks, hybrid approaches, digital twins, or in situ sensing.

Exclusion criteria: Non-peer-reviewed content (preprints, patents, theses), studies on other AM processes (DED, binder jetting), and insufficient methodological detail.

After duplicate removal and screening, 406 publications were retained, representing current PBF modeling, AI integration, and digital twin research.

**Figure 1:**
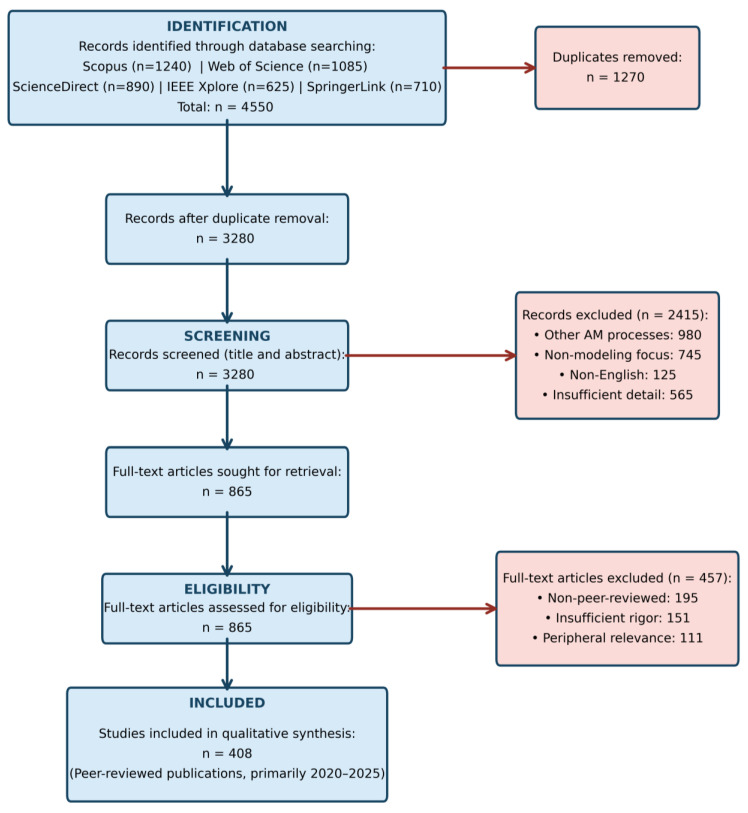
PRISMA flow diagram of study selection. Original figure created by the authors.

The screening and eligibility steps for study inclusion are detailed in the PRISMA flow diagram (Figure 1), which illustrates the number of records identified, screened, excluded, and finally included in the qualitative synthesis.

The analyzed corpus includes 408 peer-reviewed studies (2015–2025) that cover physics-based modeling, machine learning, and digital twin approaches in PBF. As shown in Table 1, LPBF dominates with titanium and nickel alloys being studied most frequently (87%). FEM and ML methods prevail, and nearly 70% of works include experimental validation.

## 2. Powder Bed Fusion in Materials Processing

The history of PBF began with the development of selective laser sintering (SLS) by Deckard at the University of Texas [13]. PBF technologies are classified into several categories: selective laser sintering (SLS), selective laser melting (SLM), direct metal laser sintering (DMLS), and electron beam melting (EBM) [14]. These processes are generally meant for rapid prototyping and rapid tooling. SLS was also known as solid freedom fabrication, layer manufacturing technology, selective laser reactive sintering, etc. [10].

Table 2 presents the major PBF variants used in additive manufacturing. Each technology offers distinct advantages and limitations based on its energy sources, processing environments, and material compatibilities.

Kruth et al. [15,16] classified PBF (SLS/SLM) processes based on the mechanisms that occur in the process into four categories:

1. Solid-state sintering: Mainly applied for consolidating ceramic powders;
2. Chemically induced binding: Not commonly used in commercial equipment, but it can be a feasible consolidation mechanism for polymers, metals, and ceramics;
3. Liquid phase sintering: Partial melting, the main mechanism in SLS for glasses, polymers, and ceramics;
4. Full melting: The basic mechanism of the SLM.

**Figure 2:**
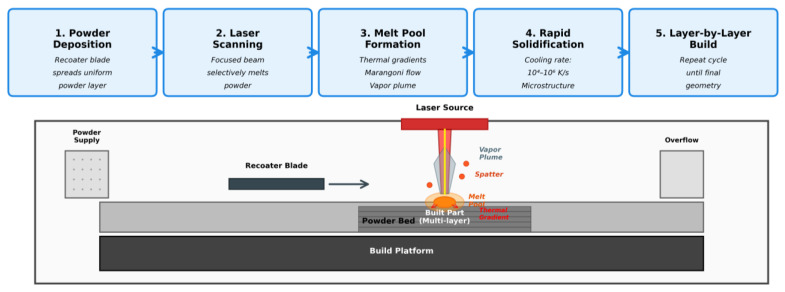
Scheme of the PBF process. Figure conceptually synthesized by the authors from widely available literature and domain knowledge.

EBM differs from other LPBF processes by the energy source (electron beam vs. laser beam) applied to metal (primarily titanium alloys) to fuse metals by melting in a vacuum environment. The basic concept of PBF is similar to that of SLA. PBF uses a moving laser or electron beam to trace and selectively sinter/melt powdered polymer and/or metal composite materials into the three-dimensional part of the final product. The product is constructed in a chamber located on a movable platform that is adjusted in height equal to the thickness of the forming layer. Powder for PBF is added from the powder feed supply, for example, with a roller. The powder is deposited on top of the powder bed in the build chamber, which contains a previously sintered or melted–solidified layer of the product and the unprocessed powder. Then the new layer is melted or sintered with a laser/electron beam. Cycles are repeated until the final product is obtained (Figure 2). SLM (and EBM) consists of four basic processes, which take place sequentially or simultaneously. They include powder deposition; heating by a laser beam and melting; free flow of molten material among unmolten and semi-melted particles and over the solid basement; and solidification. The last three processes are accompanied by heat transfer among solids, liquids, and gases, and by heat conduction.

A wide range of materials are processed in PBF [17]: aluminum alloys [19,20,21], stainless steel [22,23,24,25], Ti-6Al-4V [18,26,27,28], nickel superalloys (e.g., Inconel 718/625) [29,30,31,32], multiple materials [33,34,35], functionally graded materials (FGMs) [36,37,38], high-entropy alloys [39], and biomaterials [40,41,42]. Materials produced by SLM and EBM processing exhibit excellent static mechanical properties, equivalent to or superior to those of traditionally manufactured counterparts. However, SLS demonstrates unprocessed particles, porosity, and weakness [43,44], warping and distortion [45], limited material properties, and residual stresses [46].

Table 3 summarizes the key physical and mechanical properties (e.g., melting point, thermal conductivity, particle morphology, etc.) that influence the processability and performance of materials in PBF methods.

PBF requires numerous post-processing/finishing operations, including conventional machining, laser polishing, and electropolishing. Manufactured parts generally require material removal from surfaces to establish dimensional tolerances and achieve the desired surface properties [50].

PBF technology has several significant advantages over other additive manufacturing technologies:

- High precision and excellent surface finish: PBF processes produce parts with fine details and smoother surfaces compared to FDM or other methods without extensive post-processing, and are capable of achieving tight tolerances and intricate geometries;
- Complex geometries and internal structures: Processes are highly complex, organic, and has hollow internal features, supporting the creation of lightweight structures such as lattices and topology optimizations;
- Material diversity: Processes include high-performance alloys such as titanium, nickel-based superalloys, aluminum, and steels, enabling the production of functional, load-bearing, and wear-resistant parts in metals, as well as polymers, ceramics, glasses, and composites;
- Suitable for functional and end-use parts: The technology has high mechanical properties, enabling the rapid production of tooling, aerospace parts, medical implants, and customized components;
- Less support material required: Unlike FDM or SLA, PBF generally does not require extensive support structures because the powders themselves support overhangs and complex features during build;
- The process goes through layer-by-layer material consolidation, especially in metal PBF processes such as SLM and EBM;
- The process is capable of producing multiple parts simultaneously in a single build cycle;
- Reduced waste and material efficiency: Only the material in the powder bed is melted or sintered, so excess powder can often be recycled, reducing material waste.

PBF is a multifunctional technology that is mainly used in applications where complex geometry, material performance, and customization are crucial, such as aerospace, medical implants, automotive components, tools, jewelry, and energy. Flexibility in complex geometries and experimental manufacturing designs enables material testing, design validation, and innovative structural concepts.

In aerospace and defense applications, quality, reliability, material consumption, production costs, manufacturing time, energy consumption, occupational health, waste, impact on life cycles, capital investment, and ultimately, political issues must be considered [51]. PBF is used to manufacture engine components, turbine blades, structural components, and light lattice structures. It reduces weight and obtains complex geometry for optimized aerodynamics and a high strength-to-weight ratio. In the automotive industry, PBF is used for custom prototype parts, tools, and final parts, such as frames, exhaust components, and lightweight structures. It can be used for rapid prototyping, custom components, and lightweight components to improve efficiency [52].

In medicine and dentistry, PBF overcomes the most economic, scientific, and technical challenges. The high customization, biocompatible materials, and complex internal features allow production of personalized implants (hip, knee, dental crowns and bridges), surgical guides, and instrumentation [53,54].

In energy, PBF is utilized in energy storage and conversion systems, as well as in hydrogen reduction reactions, fuel cells, batteries, and supercapacitors [55]. It is also used in components for oil and gas, as well as parts of nuclear reactors and turbines, utilizing complex high-temperature materials and intricate cooling channels [56].

PBF is widely used for tooling and molds. Short lead times, ability to produce conformal cooling channels, and reduced costs for small batches allow the manufacturing of complex molds, inserts, and fixtures. PBF is also used in jewelry and the fashion industries. Due to the great detail resolution, complex geometries, rapid prototyping, and intricate jewelry designs, PBF produces customized accessories.

## 3. Physics-Driven Simulation Throughout the PBF Workflow

### 3.1. Powder Spreading and Packing Dynamics

Powder spreading is the foundational step in PBF that critically influences layer quality, powder bed density, and downstream defect formation. Discrete element method (DEM) simulations have emerged as the primary computational tool for modeling particle–scale interactions during recoater blade or roller spreading [57,58,59]. Advanced DEM models now incorporate realistic particle shape effects, including spherical, satellite-decorated, and irregular morphologies obtained from X-ray-computed tomography scans, which significantly affect packing density (typically 55–65% for spherical powders versus 50–58% for irregular particles) and surface roughness [57,58]. Spherical powders exhibit superior flowability characterized by Hausner ratios of 1.1–1.2, while irregular particles show Hausner ratios exceeding 1.3, indicating poor flowability and increased susceptibility to powder bed defects [58,59].

Key spreading parameters systematically investigated through DEM include recoater speed (50–500 mm/s), layer thickness (20–100 μm), blade geometry (rigid versus flexible, angle of attack), and powder flowability metrics [57,58,60]. Recent studies demonstrate that spreading speed–layer thickness combinations create distinct packing regimes: slow spreading (50–150 mm/s) with thin layers (30–50 μm) yields dense, uniform beds (ρ = 0.62–0.64) with surface roughness Ra < 3 μm but reduces productivity, while fast spreading (>300 mm/s) causes particle ejection, streaking, and density gradients (ρ < 0.55) with roughness Ra > 6 μm [57,58]. The optimal range of 100–250 mm/s balances quality and throughput for typical metal powders with particle size d_50_ = 25–45 μm [57,59].

Temperature effects introduce additional complexity—preheating the powder bed (100–200 °C for Ti-6Al-4V, 700–850 °C for electron beam melting) alters inter-particle friction coefficients, cohesion forces, and spreading dynamics through thermal expansion and incipient sintering between particles [57,59]. Coupled DEM-CFD models further capture gas flow effects during spreading, revealing that high recoater speeds generate turbulent wakes that entrain particles and create voids [57,60]. Contact mechanics parameters critical for DEM accuracy include particle–particle friction (μ_pp_ = 0.2–0.5), particle–wall friction (μ_p_w = 0.15–0.35), and coefficient of restitution (e = 0.3–0.6), typically calibrated against experimental measurements of angle of repose, flow rate, and packing density [57,58,59].

DEM challenges include computational cost (10^6^–10^8^ particles for realistic domains), parameter calibration from limited experimental data, and validation scarcity [57,58]. Multi-layer simulations incorporating thermal histories remain limited to millimeter-scale domains [57,61]. GPU acceleration, ML surrogates, and integration with melt pool models represent emerging directions for scalable predictions [57,58,59,61].

**Figure 3:**
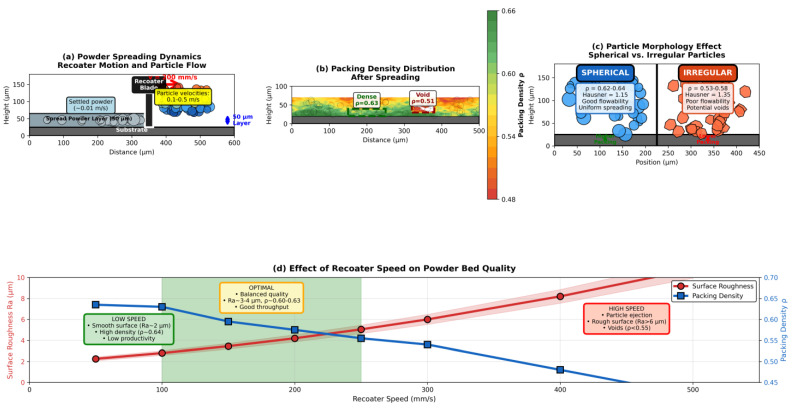
DEM simulation of powder spreading and packing behavior for Ti-6Al-4V powder: (**a**) particle velocity field, (**b**) packing density distribution, (**c**) influence of particle morphology on flowability and density, (**d**) effect of recoater speed on surface roughness and packing quality. Figure prepared using data and information reported in the cited publications.

Figure 3 demonstrates a DEM simulation of powder spreading and packing behavior for Ti-6Al-4V powder. Particle velocity field (Figure 3a) during recoater blade advancement at 200 mm/s shows active spreading zone with velocities 0.1–0.5 m/s transitioning to the settled powder bed. Packing density distribution (Figure 3b) reveals typical values of ρ = 0.60–0.63 in well-packed regions with local voids (ρ ≈ 0.51) caused by particle agglomeration or turbulent flow effects. Particle morphology (Figure 3c) influences flowability and density (spherical powders: ρ = 0.62–0.64, Hausner ratio 1.15 indicating good flowability vs. irregular particles with satellites: ρ = 0.53–0.58, Hausner ratio 1.35 indicating poor flowability). The effect of recoater speed on surface roughness and packing quality (Figure 3d) shows surface roughness increasing from Ra ≈ 2.2 μm at 50 mm/s to Ra ≈ 8.8 μm at 500 mm/s, while packing density exhibits threshold behavior, maintaining ρ ≈ 0.64 below 150 mm/s then decreasing to ρ < 0.55 above 300 mm/s due to particle ejection, with an optimal range of 100–250 mm/s for 50 μm layer thickness [57,58,59].

#### 3.1.1. Spreading Strategy Comparisons

Recoater mechanism selection—rigid blade, flexible blade, or roller—fundamentally influences powder bed quality through distinct particle interaction regimes [59,62]. Rigid blades achieve moderate packing densities (ρ = 0.58–0.62) with a surface roughness Ra = 4–7 μm and enable high speeds (>300 mm/s) but generate streaking defects on irregular substrates [63,64]. Flexible blades (elastomeric or compliant V-geometries) improve uniformity (ρ = 0.60–0.64, Ra = 3–5 μm) by conforming to substrate topography, though limited-to-medium speeds (150–250 mm/s) [62]. Rollers achieve comparable densities (ρ = 0.60–0.63, Ra = 3–6 μm) through rotational shear; counter-rotation effectively densifies cohesive powders by disrupting agglomerates, while co-rotation promotes ejection [62,65,66]. Optimal roller speeds (100–200 mm/s) balance shear-induced rearrangement against particle disturbance [67].

Key spreading mechanisms and their influence on powder layer quality are presented in Table 4.

Cohesive fine powders (d_50_ < 20 μm, Hausner > 1.3) benefit from counter-rotating rollers, while free-flowing coarse powders (d_50_ > 40 μm, Hausner < 1.2) perform adequately with rigid blades at high throughput [64,66].

#### 3.1.2. Environmental Effects: Gravity and Temperature

Gravitational acceleration profoundly affects powder dynamics, which is particularly relevant for space-based manufacturing [68]. Reducing gravity from 9.81 m/s^2^ (Earth) to 1.62 m/s^2^ (lunar, 0.17 g) decreases packing density by 8–12% and increases surface roughness by 15–20% due to reduced compaction [68]. Under microgravity (g ≈ 10^−6^ m/s^2^), cohesive forces dominate gravitational effects, causing increased particle dispersion, prolonged airborne times, and thickness variation (±30–40% versus ±5–10% at 1 g) [69]. Space applications require modified strategies: increased recoater forces, controlled gas environments, and potential electrostatic guidance [68].

Temperature significantly alters spreading through competing mechanisms [70]. Preheating from ambient (20 °C) to 100–200 °C (laser PBF) or 700–850 °C (EBM) reduces inter-particle friction (μ: 0.30–0.35 → 0.15–0.25) via surface softening, enhancing flowability [70]. However, elevated temperatures simultaneously increase cohesion through enhanced van der Waals forces, moisture condensation (<150 °C), and incipient sintering (>500–600 °C for Ti-6Al-4V, ~0.4–0.5 Tm) [70]. Non-monotonic behavior results: moderate preheating (100–150 °C) can improve spreadability, while higher temperatures (>200 °C) degrade uniformity through agglomeration, increasing density heterogeneity (CV: 6–10% → 10–15%) [70,71].

#### 3.1.3. Non-Spherical Particles and Recoater Effects

Particle morphology critically affects spreading through geometric interlocking and altered contact mechanics [72]. Spherical powders (sphericity ψ > 0.95) achieve superior flowability (Hausner 1.1–1.2), high packing density (ρ = 0.62–0.64), and low surface roughness (Ra < 3 μm), permitting aggressive spreading (250–300 mm/s) [73,74]. Irregular particles (ψ < 0.85) exhibit poor flowability (Hausner > 1.3), reduced density (ρ = 0.50–0.58), and increased roughness (Ra = 5–8 μm) due to mechanical interlocking and inefficient space-filling [72,75].

Recoater–morphology interactions amplify these effects: rigid blades encounter 2–3× higher forces with irregular powders, causing ejection and jamming [64,76]. Flexible blades partially mitigate issues through compliance but morphology effects persist [62]. Rollers show particular sensitivity—irregular particles experience increased pinching risk and fragmentation [67]. Counter-rotation partially overcomes interlocking via controlled shear but requires elevated forces [66]. Process optimization demands morphology specifications, emphasizing sphericity, parameter adjustments (reduced speed, increased force) for irregular powders, and potential conditioning (tumbling, spheroidization) [72,74].

#### 3.1.4. Packing Density Metrics and Dependencies

Packing density (solid/total volume ratio) constitutes the primary spreading quality metric, influencing melting, porosity, and mechanical properties [77,78]. Global density for spherical powders under optimal conditions ranges from 0.60 to 0.64 (approaching random close packing limit 0.64); non-spherical particles yield 0.50–0.58 [72,73]. Local density heterogeneity (CV = 5–12% for well-spread layers) increases with suboptimal parameters: high speeds (>300 mm/s) elevate CV to 15–20% via ejection/streaking [79], and cohesive powders exhibit CV 12–18% from agglomeration [71].

Measurement approaches include DEM spatial binning (high-resolution 3D mapping), transmission X-ray imaging (~10 μm resolution, 2D projection), X-ray CT (5–20 μm voxels, 3D reconstruction), and gravimetry (global only) [78,80]. Optimal density window (0.58 < ρ < 0.64) balances material availability (ρ < 0.50 causes lack of fusion) against gas permeability (ρ > 0.68 promotes gas porosity) [77,81].

The effects of powder morphology, recoating, and process conditions on layer density are presented in Table 5.

#### 3.1.5. DEM Fundamentals

The discrete element method (DEM) simulates granular systems by resolving the motion of individual particles through Newton’s equations of motion [59,73]:

Translational motion:

(1) $$m_{i}\frac{dv_{i}}{dt}=\sum bF_{ij}+F_{g,i}+F_{d,i},$$

Rotational motion:

(2) $$I_{i}\frac{d\omega_{i}}{dt}=\sum bT_{ij},$$

where $m_{i}$ is the mass of particle *i*; $v_{i}$ and $\omega_{i}$ are its translational and angular velocity vectors; $F_{ij}$ is the contact force exerted by particle *j*; $F_{g,i}$ is the gravitational force; $F_{d,i}$ is the fluid drag; $I_{i}$ is the moment of inertia; $T_{ij}$ is the torque due to contact interactions.

Contact forces are decomposed into normal and tangential components using the Hertz–Mindlin contact model [83]:

Normal force:

(3) $$F_{n}=\frac{4}{3}E^{*}\sqrt{R^{*}}\;\delta_{n}^{3/2}-\gamma_{n}v_{n,\mathrm{rel}},$$

Tangential force:

(4) $$F_{t}=\min\left(\mu F_{n},\;k_{t}\delta_{t}\right),$$

$F_{n}$ and $F_{t}$ denote the normal and tangential contact forces, respectively; $E^{*}$ and $R^{*}$ are the effective Young’s modulus and effective radius of contact; $\delta_{n}$ and $\delta_{t}$ are the normal and tangential displacements; $\gamma_{n}$ is the normal damping coefficient; $v_{n,rel}$ is the relative normal velocity; $k_{t}$ is the tangential stiffness; and μ is the coefficient of friction.

The numerical timestep is limited by the Rayleigh stability criterion:

(5) $$\Delta t\leq \frac{\pi R}{0.163\nu +0.877}\sqrt{\frac{\rho }{G}},$$

where Δt is the integration timestep, R is the particle radius, ρ is the material density, G is the shear modulus, and ν is Poisson’s ratio. For metallic powders, this typically yields Δt values on the order of $10^{-8}$–$10^{-7}$ s, requiring millions of timesteps to simulate milli-second-scale dynamics [59,61]. GPU acceleration achieves 100–1000× speed-up, enabling 10^7^–10^8^ particle simulations approaching realistic domains [61].

### 3.2. Laser–Powder Interaction and Energy Absorption

Accurate prediction of energy absorption during laser–powder bed fusion requires sophisticated ray-tracing models that account for the complex optical behavior of powder beds. The absorptivity of metal powders during LPBF processes is significantly higher (0.3–0.7 for common alloys) than that of solid surfaces due to multiple reflections and scattering between particles [84,85]. Ray-tracing simulations explicitly model laser beam propagation through the powder bed, tracking individual photon paths as they interact with particle surfaces, accounting for reflection, refraction, and absorption at each interface [84,85,86]. These models reveal that energy absorption is strongly influenced by powder morphology, particle size distribution, and packing density, with smaller particles and irregular morphologies generally increasing absorptivity through enhanced light trapping [87,88,89].

The temperature-dependent nature of absorptivity introduces additional complexity, as material optical properties evolve during heating and melting. Studies demonstrate that absorptivity increases with temperature due to changes in surface oxidation and electronic structure, necessitating dynamic updating of absorption coefficients in high-fidelity simulations. Furthermore, the transition from powder to melt pool significantly alters the absorption mechanism, with molten material exhibiting reduced absorptivity compared to the powder bed, though still higher than that of bulk solid due to surface roughness and Fresnel absorption effects. Ray-tracing models coupled with thermal simulations enable prediction of these transitions and their impact on melt pool formation, though computational cost remains a limiting factor for part-scale applications.

Absorptivity exhibits temperature dependence due to surface oxidation and changes in electronic structure, requiring dynamic updating in high-fidelity simulations [86,87]. The powder-to-melt pool transition alters absorption mechanisms, with molten material showing reduced absorptivity compared to powder beds [84,86]. Ray-tracing models coupled with thermal simulations capture these transitions, although computational cost limits part-scale applications [84,85]. Quantitative relationships between absorptivity variation and thermomechanical response are detailed in Section 3.5.2.

The powder bed characteristics exert a profound influence on laser–powder interaction and subsequent melt pool behavior. Discrete element method (DEM) simulations of powder spreading reveal that packing density varies between 0.55 and 0.65 for metal powders, with spatial heterogeneities arising from particle size distribution, spreading velocity, and layer thickness [87,88,89]. These packing variations directly affect local absorptivity and heat transfer, creating non-uniform thermal fields that influence melt pool stability and defect formation [87,88]. Particle morphology—including sphericity, surface roughness, and satellite particles—further modulates energy absorption through altered scattering patterns and inter-particle contact mechanics [87,88,89].

Experimental and computational studies demonstrate that powder bed surface roughness, typically ranging from 10 to 50 μm for common LPBF powders, affects the effective beam–material interaction area and absorption efficiency [88,89]. Irregular or agglomerated particles increase surface roughness, enhancing absorptivity but potentially introducing process instabilities through non-uniform melting [87,89]. The coupling between powder bed structure and laser absorption is particularly critical at low energy densities, where incomplete melting of poorly packed regions leads to lack-of-fusion defects [87,88]. Advanced ray-tracing models integrated with DEM-predicted powder bed geometries provide insights into these phenomena, although validation remains challenging due to the difficulty of in situ measurement of powder-scale absorption [84,85,87].

The modeling of laser–powder interaction spans multiple computational frameworks, each with distinct capabilities and limitations. Particle-based methods such as smoothed particle hydrodynamics (SPH) and the lattice Boltzmann method (LBM) naturally capture discrete powder behavior and dynamic free surfaces during melting, enabling detailed prediction of absorption and heat transfer at the particle scale [84,90,91,92]. However, these approaches face a significantly high computational cost when extended to multi-layer or part-scale simulations, often requiring simplifications in optical models or powder bed resolution [84,91]. Mesh-based finite element and finite volume methods offer computational efficiency for continuum-scale thermal analysis but struggle to represent discrete powder morphology accurately, typically resorting to effective medium approximations that may overlook local absorption heterogeneities [2,93,94].

Hybrid modeling strategies that combine ray-tracing for absorption with continuum thermal solvers represent a pragmatic compromise, enabling prediction of temperature fields with reasonable fidelity at manageable computational costs [84,92]. Model validation remains challenging due to the difficulty in directly measuring absorptivity and energy distribution during processing [86,87]. Validation typically relies on indirect metrics: melt pool dimensions, pyrometry/thermography, or post-process fusion characterization [85,86,95]. Limited experimental datasets encompassing powder bed structure, absorption, and thermal histories constrain confidence across diverse materials [84,85,86].

Table 6 highlights that model selection must balance physical fidelity with computational tractability based on the specific application. Ray-tracing models provide the highest accuracy for absorption predictions but are computationally prohibitive for routine process optimization. Effective medium approximations enable part-scale simulations but sacrifice local accuracy, making them suitable for thermal management and distortion control rather than defect-critical applications. DEM-coupled approaches bridge the powder bed structure and absorption, offering insights into the effects of powder quality but requiring careful calibration. Hybrid particle-based methods represent the state-of-the-art for fundamental studies but remain impractical for industrial deployment due to computational demands. Future advances in reduced-order modeling and machine learning surrogates may enable more efficient high-fidelity predictions, though experimental validation across diverse materials and conditions remains a persistent challenge [84,85,86]. The interplay between absorptivity variation and residual stress development is examined in Section 3.5.2, where thermomechanical coupling is addressed.

### 3.3. Melt Pool Formation and Dynamics

Melt pool formation in laser–powder bed fusion is governed by thermal–fluid interactions spanning multiple scales. High-fidelity CFD simulations incorporating energy conservation, momentum transport, and phase change provide detailed predictions [91,93,113,114]. Temperature gradients (10^4^–10^6^ K/m) and rapid solidification (10^4^–10^6^ K/s cooling rates) create transient conditions challenging numerical accuracy [113,114,115]. Surface tension gradients (∂γ/∂T ≈ −0.3 mN/m·K) generate a Marangoni convection that drives fluid from hot to cold regions, enhancing penetration and width; neglecting Marangoni effects causes up to 50% underprediction of melt pool depth [113,116,117]. At high laser power densities, recoil pressure from evaporation exceeds surface tension forces, driving keyhole formation [92,113,116,117]. While controlled keyhole melting enhances penetration, unstable keyholes collapse and trap vapor bubbles, creating porosity [86,92,113]. Advanced models incorporating these coupled phenomena successfully predict melt pool morphology and defect formation across stainless steels, titanium alloys, and nickel-based superalloys [113,115,116,117].

Analytical models (Rosenthal point/line source, Goldak double-ellipsoid) enable rapid thermal field estimation [2,114,118,119,120]. Rosenthal provides efficiency (<1 s per track) but limited accuracy (±30–50% error) by neglecting fluid flow and temperature-dependent properties [118,119,120]. Goldak improves fidelity through volumetric energy distribution (±20–30% accuracy, ~10 min per track) [2,114]. Both fail to capture keyhole formation, Marangoni flow, and defect mechanisms, requiring high-fidelity CFD with recoil pressure and vapor dynamics [92,113,116].

**Figure 4:**
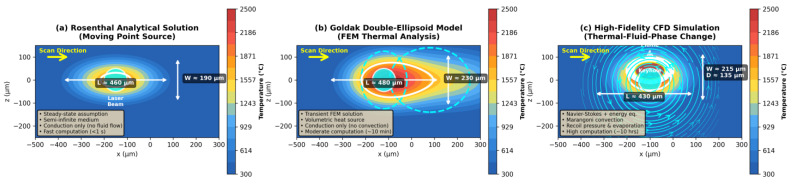
Comparison of melt pool predictions for Ti-6Al-4V using three modeling approaches: (**a**) Rosenthal analytical solution; (**b**) Goldak FEM model; (**c**) high-fidelity CFD simulation. Figure prepared using data and information reported in the cited publications.

High-fidelity multiphysics models integrate thermal, fluid, and phase change phenomena using finite volume, smoothed particle hydrodynamics (SPH), or lattice Boltzmann methods (LBMs) [84,90,91,92,113,116]. These approaches explicitly resolve the melt pool free surface and capture Marangoni convection, recoil pressure, and evaporation [91,92,113,116]. Despite detailed physics, many models simplify or omit powder-scale effects and dynamic powder bed interactions [91,121]. The treatment of vaporization and keyhole dynamics remains challenging due to complex multi-phase interactions and rapid transient phenomena [84]. The trade-off between computational efficiency and physical realism (Figure 4) guides model selection: Rosenthal/Goldak is needed for parameter screening and process mapping across large design spaces; CFD is needed for defect-critical applications requiring ±10–20% accuracy in melt pool predictions [2,8,9,114].

Extreme conditions and rapid time scales complicate a validation of melt pool models. High-speed imaging, pyrometry, and X-ray radiography provide valuable data but face limitations in spatial and temporal resolution [86,95,122]. High-speed cameras capture melt pool surface dynamics at frame rates exceeding 10^4^ Hz, enabling observation of keyhole oscillations and spatter ejection [86,115]. Pyrometry and infrared thermography measure surface temperature distributions but are affected by emissivity variations and calibration challenges [95,123,124]. Synchrotron X-ray imaging offers unprecedented access to subsurface melt pool dynamics and keyhole formation, but is limited to specialized facilities [86]. Post-process characterization via optical microscopy and X-ray computed tomography provides detailed information on melt pool geometry and defect distribution but lacks temporal resolution [115,125]. The scarcity of comprehensive validation datasets encompassing multiple materials, process parameters, and measurement techniques limits confidence in model predictions [1,8,9].

Table 7 emphasizes that no single modeling approach satisfies all requirements for accuracy, computational efficiency, and physical completeness. Analytical models enable rapid parameter screening but lack predictive capability for defects. Continuum FEM/FVMs offer a practical balance for part-scale predictions but may miss critical fluid flow effects. High-fidelity CFD offers the most accurate melt pool predictions but remains computationally prohibitive for routine use. Particle-based methods excel at capturing powder-scale physics but face severe scalability limitations. Reduced-order models show promise for bridging fidelity and efficiency, although their reliability depends critically on the quality of training data. Future progress requires multi-scale frameworks that strategically deploy high-fidelity simulations where needed while leveraging efficient approximations elsewhere, guided by rigorous experimental validation [8,9,113,114].

### 3.4. Solidification and Microstructure Evolution

PBF processes are complex AM techniques that require precise modeling of microstructure evolution, grain growth dynamics, and phase transitions. Two prominent computational models used for these purposes are the phase-field (PF) and cellular automaton (CA) models.

Phase-field models are highly versatile and accurate for simulating microstructure evolution during PBF processes. They solve physical equations that govern phase transitions and grain growth, making them particularly effective in capturing detailed microscopic characteristics such as dendritic growth, stability of the solid–liquid interface, and grain nucleation [142,143,144]. For example, a three-dimensional phase-field model was used to simulate grain evolution during a three-layer, three-track PBF process, reproducing nucleation, growth, and coarsening processes in melting pools and heat-affected zones [144]. Similarly, phase-field models have been applied to study competitive growth and nucleation in alloys with spatially varying initial compositions, revealing the influence of composition on dendrite morphology [142].

Phase-field models are widely used in both academic research and industrial applications. They provide insight into the relationship between process parameters (e.g., laser power and scan speed) and microstructure evolution, allowing optimization of PBF processes [142,145]. For example, a nonisothermal phase-field model was used to generate a densification map for 316L stainless steel, classifying the process parameters based on the resulting morphologies [145]. A transient 3D multi-scale phase-field model was developed to simulate microstructure evolution in Ti-6Al-4V components produced by LPBF. The model accounted for rapid solidification conditions and the impact of process parameters (e.g., laser power, speed, beam shape) on grain morphology and texture. The results were validated against experimental data, demonstrating the ability of the model to predict grain size and texture evolution [49]. In addition, phase-field models have been coupled with machine learning techniques to accelerate simulations, allowing large-scale predictions of the microstructure evolution in PBF processes [146,147].

Despite their accuracy, phase-field models suffer from high computational costs, limiting their application to large-scale simulations relevant to AM processes. To address this, researchers have developed surrogate machine learning models trained in small-scale phase-field simulations, reducing computational time by orders of magnitude while maintaining high accuracy [146,147].

Cellular automaton (CA) models are computationally efficient tools for simulating microstructure evolution and grain growth in PBF processes. They use a grid-based approach with predefined rules to model nucleation and growth, making them suitable for large-scale simulations [148,149,150]. For example, a coupled CA-FE model was developed to predict grain texture evolution during the solidification of Alloy 625, capturing competitive grain growth and columnar/equiaxed grain structures [149]. Similarly, a 3D CA model was used to simulate the evolution of grain structure in multi-layer, multi-track LPBF components, considering factors such as thermal gradients and epitaxial growth [151,152].

Cellular automaton models are particularly useful for studying the effects of process parameters on grain morphology and texture. For example, a CA model was used to investigate the dependence of microstructure on the printing areas of Inconel 625, revealing a weaker texture intensity and more heterogeneous grain structures in broader sections [148]. Additionally, CA models have been coupled with finite element models to simulate the thermal history of PBF processes, allowing predictions of grain nucleation and growth based on temperature fields [149,153].

Although CA models are computationally efficient, they may lack the resolution to capture detailed microscopic features such as transient growth conditions and stability of the solid–liquid interface, which are critical for accurate microstructure prediction [144]. However, refinements to the CA grid and the introduction of additional physical considerations, such as thermal gradients and solid–solid interface growth, have improved the flexibility and accuracy of these models [148].

**Figure 5:**
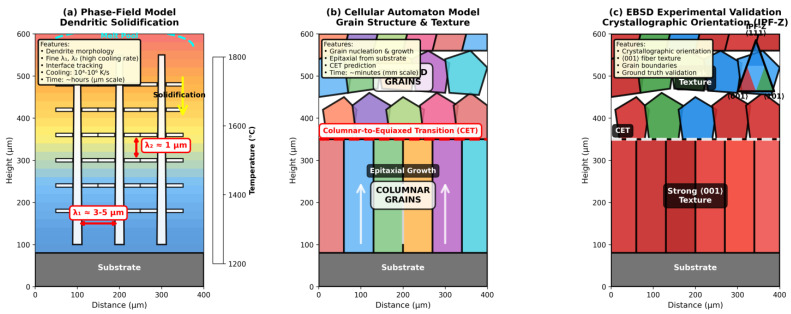
Integration of phase-field and cellular automaton methods for microstructure evolution modeling, validated by EBSD data: (**a**) phase-field simulation captures dendritic arm spacing under LPBF conditions; (**b**) CA model predicts grain structure and columnar-to-equiaxed transition; (**c**) EBSD map confirms ⟨001⟩ texture and transition zone. Figure prepared using data and information reported in the cited publications.

Figure 5 illustrates the complementary roles of phase-field and cellular automaton methods in predicting microstructure evolution, validated against experimental EBSD measurements. Phase-field simulations (Panel A) resolve dendritic solidification at the interface scale, capturing primary dendrite arm spacing (λ_1_ = 3–5 μm) and secondary arm spacing (λ_2_ ≈ 1 μm) characteristic of LPBF’s high cooling rates (10^4^–10^6^ K/s). These fine-scale features contrast sharply with conventional casting (λ_1_ = 50–100 μm at ~10^2^ K/s), reflecting the relationship λ_1_ ∝ (cooling rate)^−0.5^. Cellular automaton models (Panel B) operate at the mesoscale to predict the evolution of the grain structure, epitaxial growth from the substrate, and the location of the columnar-to-equiaxed transition (CET). The CET typically occurs at 200–400 μm above the substrate, where the thermal gradient-to-solidification velocity ratio (G/R) decreases sufficiently to favor equiaxed nucleation. EBSD validation (Panel C) confirms strong ⟨001⟩ fiber texture in the columnar region (red IPF-Z coloring) due to preferential grain growth along the thermal gradient direction, with texture strength diminishing in the equiaxed region. Quantitative comparisons show that phase-field predictions achieve ±15–25% accuracy for dendrite arm spacing, while CA models predict grain size distributions within ±20–30%, of which both are validated with experimental measurements [139,142,143,148,149,151,152,153,154,155].

### 3.5. Thermomechanical Response and Residual Stress

Thermomechanical modeling constitutes a critical component of powder bed fusion (PBF) process simulation, enabling prediction of residual stresses, thermal gradients, and part distortion that directly influence dimensional accuracy and mechanical integrity [2,156]. Finite element method (FEM) simulations incorporating sequentially coupled thermal–mechanical analyses capture the complex interplay between rapid heating and cooling cycles, temperature-dependent material properties, and evolving stress states across multiple length scales [94,157]. These models typically employ element activation techniques to simulate layer-by-layer material deposition, nonlinear thermoelastoplastic constitutive laws to represent material behavior under extreme thermal gradients (exceeding 10^6^ K/s), and adaptive meshing strategies to balance computational efficiency with spatial resolution requirements [158,159].

Inherent strain methods offer computationally efficient alternatives by extracting effective plastic strains from detailed process simulations and applying them at the part scale, achieving computational speed-ups of 6–100× while maintaining prediction accuracy within 10–15% for residual stress and distortion [160,161]. Modified inherent strain approaches incorporate temperature-dependent mechanical properties, anisotropic effects induced by scanning strategies, and shear strain components to enhance fidelity [162,163,164]. Validation against experimental measurements, including synchrotron diffraction, neutron diffraction, X-ray diffraction, and optical three-dimensional scanning, consistently demonstrates that both FEM and inherent strain frameworks capture residual stress distributions and distortion patterns with errors typically below 15%, although computational cost–accuracy trade-offs remain significant [165,166,167].

#### 3.5.1. Influence of Preheating on Residual Stress

Substrate and powder bed preheating represents a widely adopted strategy for mitigating residual stress and distortion in PBF by reducing thermal gradients during processing [166]. Thermomechanical simulations demonstrate that preheating Ti-6Al-4V substrates from ambient temperature (20 °C) to elevated temperatures (200–300 °C) reduces peak thermal gradients by 30–50%, corresponding to proportional reductions in maximum residual stress magnitudes of 40–60% [156,168]. The mechanism underlying stress reduction involves decreased temperature differentials between molten material and surrounding solid regions, resulting in lower thermal strain accumulation during solidification and reduced driving force for plastic deformation [166].

The temperature-dependent yield strength governs stress evolution during thermal cycling and can be approximated by the following:

(6) $$\sigma_{y}\left(T\right)=\sigma_{y0}-k\;T$$

where $\sigma_{y0}$ is the yield strength at the reference temperature (Pa), k is the thermal softening coefficient (typically 0.5–1.5 MPa·K^−1^ for Ti–6Al–4V), and T is the temperature rise above the reference state (K) [156]. At elevated preheating temperatures, reduced yield strength facilitates stress relaxation through plastic flow, preventing accumulation of elastic residual stresses that would otherwise persist upon cooling to ambient conditions [169].

Preheating also redistributes tensile and compressive stress zones: without preheating, high tensile stresses concentrate near scan track edges and top surfaces (often exceeding 500–700 MPa for Ti-6Al-4V), while compressive stresses develop in substrate regions adjacent to the build [157,170]. With 200 °C preheating, peak tensile stresses reduce to 300–400 MPa, and the compressive zone extends deeper into the substrate, improving stress balance and reducing distortion tendency [156,168]. However, excessive preheating (>400 °C for Ti alloys) can promote grain coarsening, alter phase transformation kinetics, and reduce mechanical property uniformity, necessitating optimization of preheating temperature for specific material–geometry combinations [166].

#### 3.5.2. Role of Absorptivity Variation

Laser absorptivity variation (A = 0.3–0.7 for metallic powders, detailed in Section 3.2) critically influences energy coupling efficiency, melt pool dimensions, and thermal gradients driving residual stress formation [156,171,172]. Temperature-dependent absorptivity (increasing with electron–phonon coupling) is calculated as follows [156,173]:

(7) $$A\left(T\right)=A_{0}+\beta \;T,$$

where *A*_0_ is the baseline absorptivity; β is the temperature coefficient of absorptivity (typically 1–3 × 10^−4^ K^−1^), quantifying the thermal sensitivity of absorptivity; and *T* is the temperature (or temperature rise) in kelvin (K).

Absorptivity variations of ±20% translate to melt pool depth fluctuations of ±20–30% and width variations of ±10–15%, directly affecting fusion quality and residual stress [171,172]. Higher absorptivity enlarges melt pools, intensifies thermal gradients, and increases residual stress by 15–25%; lower absorptivity reduces penetration, risking lack-of-fusion defects [156,171].

The sensitivity of residual stress predictions to absorptivity underscores the importance of accurate material characterization and considering surface condition evolution during multi-layer builds [172]. Powder layers exhibit higher absorptivity (0.4–0.6) than solidified surfaces (0.3–0.45) due to multiple scattering and cavity effects, creating layer-dependent energy coupling that influences thermal history and stress accumulation [156,173]. Advanced thermomechanical models incorporate temperature- and state-dependent absorptivity to capture these effects, improving prediction accuracy for residual stress and distortion [171].

#### 3.5.3. Combined Process Parameter Interactions

Laser power (P), scan speed (v), and hatch spacing (h) constitute the primary process parameters governing energy density (*E* = *P*/(*v*·*h*·*t*), where *t* is layer thickness) and resulting in the thermal–mechanical response in PBF [156,174]. These parameters exhibit strong nonlinear interactions: increasing laser power from 150 W to 250 W while maintaining constant scan speed (800 mm/s) and hatch spacing (0.1 mm) elevates peak temperatures by 200–300 K, expands melt pool volume by 40–60%, and increases maximum residual stress by 25–35% [156,168]. However, simultaneous increases in scan speed proportionally reduce energy density, partially offsetting power effects and creating complex parameter coupling [174].

Response surface methodology (RSM) and factorial design studies reveal that scan speed exerts the strongest influence on residual stress magnitude (accounting for 40–50% of variance), followed by laser power (30–35%) and hatch spacing (15–20%) [172,174]. Interaction effects between parameters account for an additional 10–15% of variance, emphasizing the inadequacy of one-factor-at-a-time optimization approaches [174]. For example, the stress-reducing benefit of increased scan speed diminishes at low laser powers where insufficient fusion occurs, while high-power, high-speed combinations can induce keyhole porosity that locally concentrates stresses [156].

Hatch spacing influences residual stress through its effect on thermal overlap between adjacent scan tracks: smaller spacing (0.08–0.10 mm) increases re-melting and reheating of previously solidified material, promoting stress relaxation but also elevating cumulative heat input and part-scale thermal gradients [168,172]. Larger spacing (0.12–0.15 mm) reduces inter-track thermal interaction, potentially decreasing global residual stress but risking lack-of-fusion defects between tracks that act as stress concentrators [172,174]. Optimal parameter combinations identified through thermomechanical simulations typically balance energy density near the material-specific threshold for full melting (60–80 J/mm^3^ for Ti-6Al-4V) while minimizing thermal gradients through moderate power (180–220 W) and speed (900–1100 mm/s) selections [156,168].

#### 3.5.4. Laser Operation Mode Effects

Continuous wave (CW) versus pulsed laser operation modes fundamentally alter temporal energy delivery, resulting in a thermal–mechanical response in PBF [169,171]. CW lasers maintain constant power output throughout scanning, generating steady-state melt pools with relatively uniform thermal gradients along the scan direction. Pulsed mode operation introduces periodic energy modulation (pulse duration 0.1–10 ms, duty cycle 50–90%), creating transient melt pool dynamics with alternating heating and partial cooling phases [171].

Thermomechanical simulations demonstrate that pulsed laser operation with optimized parameters (pulse duration 2–5 ms, duty cycle 70–80%, peak power 1.5–2× average CW power) reduces the maximum residual stress by 20–40% compared to equivalent average-power CW processing [169]. The stress reduction mechanism involves intermittent heating that allows partial stress relaxation during pulse-off intervals, reducing peak thermal gradients (by 15–25%) and limiting plastic strain accumulation [171]. Additionally, pulsed operation promotes finer microstructures through repeated thermal cycling, potentially enhancing mechanical properties [169].

However, pulsed mode introduces fusion quality risks: insufficient pulse overlap causes periodic lack-of-fusion defects between pulses, while excessive peak powers during pulse-on phases can induce keyholing and porosity [171]. Optimal pulsed operation requires careful matching of pulse frequency to scan speed to ensure adequate melt pool overlap (typically 60–80% overlap between consecutive pulses) [169]. Modulated power strategies employing gradual power ramping at scan track start/end or layer transitions offer intermediate approaches that reduce residual stress (by 10–20%) while maintaining fusion integrity superior to aggressive pulsing [158,169].

The computational modeling of pulsed laser operation demands finer temporal resolution (timesteps 0.1–1 ms versus 5–10 ms for CW) to capture transient thermal dynamics, increasing simulation cost by factors of 5–10 [171]. Hybrid models employing detailed pulsed simulations for calibration followed by effective continuous heat source representations at the part scale offer practical compromises for design optimization [169].

The influence of key process parameters on residual stress and distortion is summarized in Table 8.

**Figure 6:**
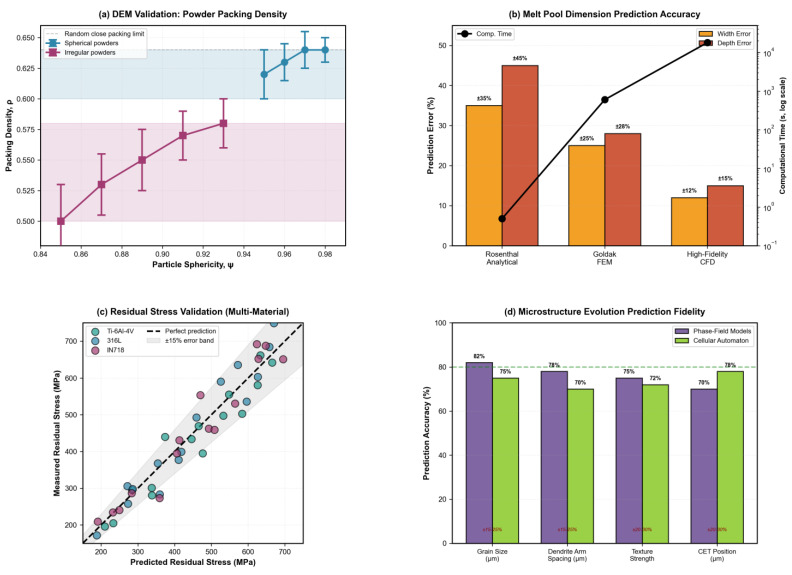
Validation of PBF modeling accuracy and efficiency across the full modeling chain: (**a**) DEM powder packing accuracy [57,58,72,73], (**b**) melt pool cost–accuracy trade-offs across analytical, FEM, and CFD models [2,121,122], (**c**) thermomechanical residual stress validation [145,146,154,155,156], and (**d**) microstructure prediction fidelity of phase-field and cellular automaton methods [128,131,132,137,138,140,141,142], illustrating the balance between physics fidelity and computational cost. Figure prepared using published data.

Figure 6 presents an integrated view of validation metrics across the entire PBF modeling chain, illustrating how accuracy and efficiency evolve from powder to part scale. Starting with DEM simulations, the models achieve 5–10% accuracy in predicting powder packing density for well-characterized morphologies, forming a reliable basis for subsequent melt pool modeling. Melt pool simulations reveal the steep computational trade-offs among analytical, finite element, and high-fidelity CFD approaches—spanning sub-second to hour-long runtimes and errors from ±30–45% down to ±10–15%. At the part scale, thermomechanical predictions align with synchrotron and neutron diffraction data within ±10–15% for key alloys such as Ti-6Al-4V, 316L, and IN718. Finally, microstructure modeling shows phase-field methods attaining 70–82% accuracy for grain and dendrite features, while cellular automaton models achieve 70–78% with greater efficiency. Together, these results underscore the central challenge of balancing physical realism with computational tractability in advancing PBF modeling toward industrial application.

## 4. Evolution of Modeling Approaches

The modeling of powder bed fusion (PBF) has undergone a remarkable evolution, reflecting the increasing complexity and industrial relevance of the process. Early work relied on empirical correlations and analytical heat transfer models, which provided computationally efficient tools for mapping process parameters and defect regimes, but suffered from oversimplifications that limited predictive accuracy. With advances in computational power, physics-based numerical simulations emerged, enabling multiphysics and multi-scale insights into thermal fields, melt pool dynamics, powder spreading, and residual stress formation. More recently, hybrid modeling strategies have sought to combine the rigor of physics-based approaches with the adaptability of data-driven and machine learning methods, improving both predictive fidelity and computational efficiency. Complementing these developments, the integration of in situ monitoring data has opened new possibilities for real-time calibration, adaptive control, and the eventual realization of digital twin frameworks. Together, these advances chart a trajectory from simple analytical tools toward intelligent, data-integrated models capable of guiding defect mitigation, microstructure control, and performance optimization in PBF.

**Figure 7:**
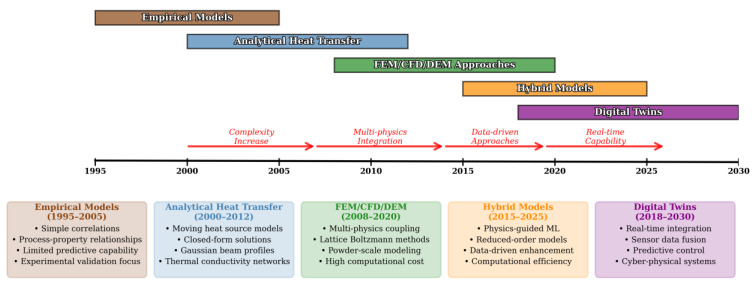
Timeline of PBF modeling evolution. Figure conceptually synthesized by the authors from the widely available literature and domain knowledge.

Figure 7 outlines the progression of PBF modeling methods, from early empirical approaches to advanced digital twin frameworks.

### 4.1. Physics-Based Modeling

The integration of empirical data with analytical solutions enhanced the robustness of process maps and improved defect prediction frameworks [119,178,179]. Dimensionless parameters and scaling laws were introduced to generalize processing windows, yet the static nature of many early models hindered their application in dynamic process control and real-time optimization [47,180]. Critical assumptions—including uniform powder bed properties, simplified heat source representations, and steady-state conditions—reduced physical realism and limited accurate prediction of process instabilities under practical manufacturing conditions [47,181,182].

Physics-based numerical simulations address the limitations of analytical models through detailed multiphysics representations. Mesh-based methods (FEM, FVM) provide validated accuracy for temperature distributions, residual stresses, and distortions [1,2,93], though challenges persist in handling large deformations and fine-scale melt pool dynamics [2,183].

Particle-based methods—including discrete element method (DEM), smoothed particle hydrodynamics (SPH), and lattice Boltzmann method (LBM)—offer flexible frameworks for modeling discrete powder behavior, melt pool fluid dynamics, and complex free surface phenomena [1,59,60,84,91,184]. GPU acceleration and adaptive refinement techniques have enabled mesoscale simulations with improved resolution, though computational intensity remains a barrier to routine application [61,90,185]. Coupled approaches, such as DEM-CFD, DEM-LBM, and DEM-SPH, have demonstrated enhanced predictive capabilities for powder–fluid interactions, vapor–powder–melt dynamics, and microstructure evolution [60,101,186,187]. Cellular automata (CA) coupled with LBM have effectively simulated powder dynamics and thermal–fluid interactions [188].

Despite these advances, multiphysics integration increases model complexity and computational cost, often necessitating simplifications that may compromise accuracy [1,106]. The validation of coupled models is challenging due to limited comprehensive experimental data spanning all relevant phenomena [101,187]. Parameter sensitivity, numerical stability, and scalability issues further constrain the robustness and industrial applicability of advanced numerical frameworks [60,186]. Mesh-based methods struggle with accurate representation of discrete powder behavior and dynamic free surfaces, while mesh-free methods face challenges in numerical stability and parameter calibration [1,59,102,106].

The transition from empirical to physics-based modeling reflects a broader shift toward higher-fidelity, multi-scale approaches capable of capturing the intricate interplay of thermal, fluid, and mechanical processes in PBF.

Table 9 synthesizes the principal capabilities, computational efficiency, and limitations across this modeling spectrum, highlighting the critical trade-offs that continue to shape model selection and development priorities.

The synthesis reveals that while empirical and analytical models remain valuable for rapid process design and parameter screening, the increasing demand for predictive accuracy and physical realism has driven adoption of computationally intensive physics-based simulations. The ongoing challenge lies in balancing model fidelity with computational feasibility, particularly for industrial-scale applications requiring real-time or near-real-time process optimization and control.

### 4.2. Hybrid Physics–Data-Driven Approaches

The convergence of physics-based simulations and data-driven techniques addresses a fundamental tension in PBF modeling: physics-based models require prohibitive computational resources for real-time control, while purely data-driven approaches lack physical interpretability and generalizability beyond training domains [204,205]. Hybrid frameworks leverage complementary strengths—physics components encode governing equations ensuring thermodynamic consistency, while machine learning accelerates computations and identifies latent patterns in complex process signatures [204,205,206]. This synergy is critical for PBF, where rapid thermal cycles, stochastic powder bed heterogeneity, and transient melt pool dynamics generate high-dimensional process spaces, challenging both purely analytical and empirical methods [205].

Recent hybrid efforts demonstrate substantial progress in melt pool prediction and thermal history estimation. Integration of CFD with machine learning improves melt pool width prediction accuracy while reducing computational time [204]. Physics-informed neural networks (PINNs) embed governing equations into neural network loss functions, enabling temperature field prediction with limited training data [205]. These frameworks consistently outperform standalone approaches in predicting melt pool dynamics, thermal profiles, and defect formation [111,204,205], with experimental validations supporting reliability [205,206]. Concurrent modeling of microstructural evolution and porosity enhances practical relevance [111], while techniques such as equivalent boundary condition methods optimize computational efficiency [158].

Hybrid model applicability spans stainless steels, titanium alloys, aluminum alloys, and multi-material systems [206,207,208]. Integration with digital twin frameworks supports defect prediction, parameter optimization, and adaptive control in aerospace, biomedical, and defense sectors [7,209,210,211]. Hybrid manufacturing strategies that combine LPBF with milling or direct energy deposition expand application scope [212,213,214], demonstrating improvements in surface finish, dimensional accuracy, and mechanical performance [214,215].

Despite progress, significant challenges constrain broader adoption. Integration methods lack standardization, and the fusion of heterogeneous data sources introduces biases [204,205]. Heavy reliance on extensive datasets or high-fidelity simulations limits generalizability across alloys and process regimes [204,205,207,208,216]. Validation remains constrained by material-specific campaigns, restricting transferability to novel systems [208,216]. Computational efficiency gains have not yet enabled real-time control for full-component simulations with detailed multiphysics [111,205]. Scalability challenges persist in extending models to industrial applications, requiring real-time data acquisition and integration with manufacturing execution systems [7,217]. Translation to industrial practice is hindered by challenges in model updating and integration with legacy systems [7,217], while optimization of hybrid process parameters remains complex and often empirical [207,208,214].

Table 10 synthesizes principal advances and persistent challenges.

Hybrid physics–data-driven approaches represent a promising yet still-maturing paradigm. While substantial progress has been achieved in prediction accuracy and computational efficiency, realizing full potential for industrial-scale, real-time process control requires continued advances in data integration, model standardization, multi-scale coupling, and experimental validation across diverse material systems and manufacturing conditions.

### 4.3. Integration of In Situ Monitoring Data

The literature shows notable progress in leveraging in situ thermal, optical, and multi-sensor data for calibrating PBF models, enhancing process insight, and defect prediction. Many studies demonstrate the potential of machine learning techniques to enhance defect detection and process control, while others focus on the challenges of data registration and sensor fusion. However, limitations persist in the robustness of methodologies, data quality, and the generalizability of models across different geometries and process conditions. The synthesis underscores the need for improved calibration protocols, comprehensive multi-modal data integration, and real-time adaptive control strategies to fully leverage in situ monitoring for PBF model calibration. Table 11 provides a systematic, evaluative assessment of in situ monitoring approaches for powder bed fusion, addressing sensor capabilities, validation rigor, computational performance, transferability, and industrial readiness. Each entry identifies which methods are most credible for specific purposes.

## 5. Digital Twins and AI Integration for PBF

Digital twins represent the convergence of physics-based modeling, real-time sensing, and artificial intelligence into integrated frameworks for PBF process control and optimization. This section examines the architectural foundations of PBF digital twins, the multi-modal data streams that enable them, the AI methods that power real-time inference, and industrial implementations that demonstrate their capabilities.

### 5.1. Digital Twin Concept and Architecture

A digital twin (DT) for PBF comprises three interconnected architectural layers that enable real-time process monitoring and control [251].

The data layer integrates multi-modal sensor fusion, combining optical, thermal (infrared), acoustic emission, and pyrometry data through feature-level or decision-level fusion strategies. High-speed multi-modal systems achieve 95–98.5% accuracy in flaw detection with temporal resolution up to 20 kHz, proposed for intra-layer closed-loop control [252,253].

At the foundational level, the digital twin begins with high-fidelity data acquisition from the physical PBF system. The PBF process involves intricate interactions among laser energy input, powder feedstock behavior, thermal gradients, and environmental conditions. Capturing these phenomena requires a robust sensor suite, including photodiodes, infrared pyrometers, high-speed cameras, and thermocouples [254]. These sensors monitor key parameters, including melt pool geometry, temperature distributions, powder flow consistency, and layer integrity. The sensor data is typically generated at high frequencies and in large volumes, which necessitates efficient edge computing strategies for real-time pre-processing. Utilizing edge devices mitigates latency and data bottlenecks while ensuring time-critical information is delivered to higher digital layers with minimal delay [255].

Once collected, sensor data is channeled through standardized communication protocols, such as OPC-UA or MQTT, into centralized data repositories. These repositories organize information as time-series records and metadata registries, capturing everything from machine configurations to build plans and environmental variables. This layer supports data provenance and traceability—an essential requirement for certification in regulated industries. Furthermore, the integration of machine and process metadata into a unified data framework enables correlation of in-process signals with final part quality metrics.

The physics layer employs reduced-order models, surrogate models, or hybrid computational fluid dynamics (CFD)/finite element method (FEM) simulations to predict thermal history, melt pool dynamics, and microstructure evolution. Bayesian calibration frameworks enable probabilistic DTs that quantify uncertainty and support layer-wise parameter adjustment based on real-time sensor feedback [256].

The modeling and simulation layer builds on this data foundation by providing a virtual mirror of the PBF process. High-fidelity finite element and computational fluid dynamics (CFD) models simulate heat transfer, melt pool behavior, phase transitions, and residual stress evolution during fabrication. These physics-based models are invaluable for predicting defect formation, including porosity, keyholing, and delamination. However, due to their computational intensity, they are often supplemented by surrogate models—typically built using machine learning techniques—that approximate system behavior with significantly lower latency. Notably, deep neural operators and Fourier neural operator frameworks have been proposed to emulate temperature fields and melt pool dynamics in real time, allowing predictive control and uncertainty quantification [224].

The AI layer provides rapid inference through trained ML models, with potential for predictive maintenance, defect prognosis, and adaptive control at latencies approaching process time scales under controlled experimental conditions [8].

The analytics and inference layer acts as the digital twin’s decision engine. This component employs machine learning models, statistical algorithms, and signal processing tools to extract actionable insights from process data. It identifies anomalies, estimates quality outcomes, and recommends corrective measures. For instance, supervised learning algorithms can classify melt pool instabilities, while unsupervised models detect deviations from baseline operation. The control module then used these predictions to enact dynamic adjustments in process parameters. Closed-loop feedback control, based on such analytics, has been demonstrated to stabilize the process by actively adjusting laser power or scan speed in response to emerging anomalies in controlled experimental studies on specific materials and machine configurations [257,258].

Real-time synchronization between physical and virtual models is achieved through bidirectional data exchange, where sensor measurements continuously update the virtual representation while simulation predictions guide the adjustments to the physical process [7].

Human interaction with the digital twin is facilitated through advanced visualization and user interface systems. Real-time dashboards and 3D process visualization tools offer intuitive representations of part progress, thermal maps, and defect probabilities. Augmented reality (AR) interfaces further enhance operator situational awareness by overlaying digital process data onto physical environments, streamlining maintenance and inspection workflows [259,260]. These visualizations are not merely cosmetic; they are critical for diagnostics, decision-making, and collaboration across multidisciplinary teams.

For comprehensive functionality, the digital twin must also be seamlessly integrated into enterprise IT systems. Integration with product lifecycle management (PLM), manufacturing execution systems (MESs), and enterprise resource planning (ERP) platforms enables the twin to access design intent, monitor production workflows, and link to supply chain logistics. This enterprise connectivity transforms the digital twin into a strategic asset that spans design, production, and business decision layers [261,262].

**Figure 8:**
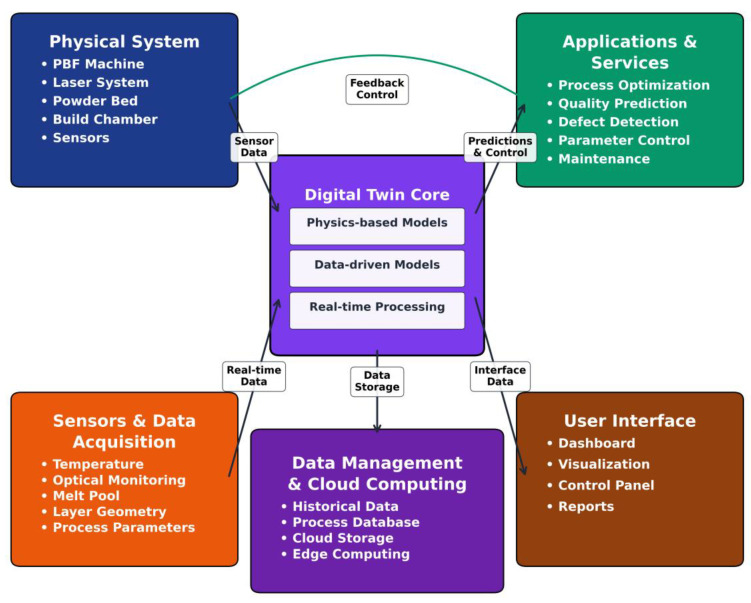
Ecosystem of a PBF digital twin. Figure conceptually synthesized by the authors from widely the available literature and domain knowledge.

Figure 8 illustrates the complete ecosystem of a PBF digital twin.

### 5.2. Data Sources and Sensor Integration

Sensor technologies and data integration methods form the foundation of PBF digital twin development. Recent advances in thermography, pyrometry, acoustic monitoring, and melt pool imaging enable multi-sensor fusion for defect detection and process control. Challenges persist in spatial–temporal data registration, signal interpretation, and the implementation of FAIR data principles, with gaps remaining in standardization and industrial scalability.

Table 12 summarizes sensor technologies and data fusion approaches for PBF digital twins, evaluating specifications, validation, computational performance, transferability, and industrial readiness.

**Figure 9:**
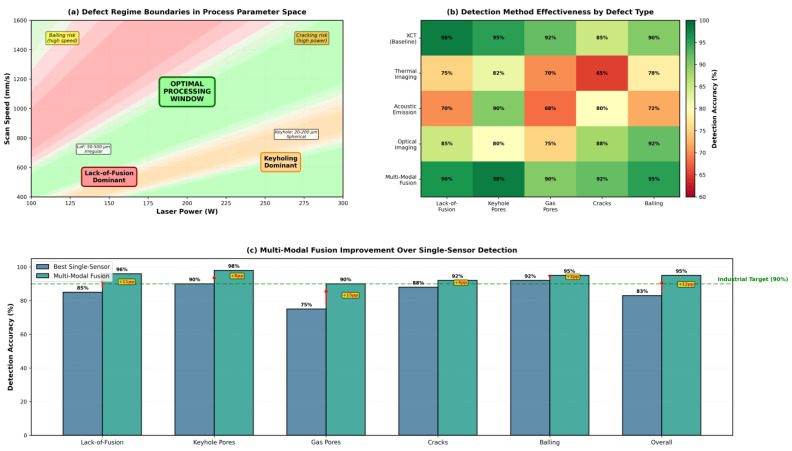
Defect formation and detection in PBF: (**a**) process maps showing defect regimes for Ti-6Al-4V [4,51,86,91,92,113,116,274,275,276,277], (**b**) detection accuracy of thermal, acoustic, optical, and XCT methods across defect types [278,279,280,281,282,283,284,285,286], and (**c**) multi-sensor fusion gains improving overall accuracy to 95% and meeting industrial reliability thresholds [278,279,280,281,285]. Figure prepared using published data.

Figure 9 summarizes defect regimes, detection performance, and sensor fusion gains across the major PBF defect types. Distinct process windows in Ti-6Al-4V delineate lack of fusion at low energy densities, keyhole porosity at high energies, and an optimal zone minimizing both. Detection benchmarking shows X-ray CT as the validation reference, with optical and acoustic sensors excelling at surface and transient defects, respectively, while thermal imaging captures energy coupling variations. Multi-modal fusion combining these signals improves overall accuracy from 83% to 95% and boosts defect-specific detection—especially for gas porosity and lack of fusion—by exploiting complementary sensing physics. These results confirm that integrated sensor fusion surpasses the 90% accuracy threshold required for reliable, real-time defect classification in digital twin-enabled PBF systems.

### 5.3. AI and Machine Learning Methods

Artificial intelligence (AI) and machine learning (ML) have emerged as transformative paradigms for modeling and controlling powder bed fusion (PBF) processes, addressing the inherent complexity of multiphysics phenomena that govern part quality and process stability [280]. PBF involves rapid thermal cycling, complex melt pool dynamics, and stochastic defect formation mechanisms that are difficult to capture through physics-based models alone [112]. Data-driven ML methods complement traditional computational approaches by learning complex nonlinear mappings from high-dimensional sensor data to process outcomes, enabling predictions of melt pool geometry, thermal history, and defect formation [5]. The integration of AI into PBF process chains has been proposed to facilitate real-time decision-making, adaptive parameter control, and quality assurance, bridging the gap between laboratory-scale understanding and industrial deployment [287].

#### 5.3.1. Supervised Learning for Defect Detection

Supervised learning forms the foundation of most ML applications in PBF, where labeled datasets relating process parameters to outcomes enable predictive modeling. Convolutional neural networks (CNNs) have demonstrated very high accuracy in defect detection from optical and thermal imagery, classifying porosity, lack of fusion, and surface anomalies in real-time layer-wise monitoring [281]. Ensemble methods such as random forest and XGBoost enable online process monitoring [281]. Transfer learning extends model applicability across materials, with cross-material quality monitoring achieving >92% accuracy after fine-tuning with limited target domain data [288].

#### 5.3.2. Self-Supervised and Semi-Supervised Learning

Self-supervised learning addresses the challenge of unlabeled data in PBF through clustering and anomaly detection. Self-supervised learning frameworks reduce labeling requirements by leveraging temporal correlations in high-speed video data, achieving multi-label melt pool anomaly classification without extensive manual annotation [289]. Semi-supervised approaches combine limited labeled data with abundant unlabeled observations to improve model generalization, which is particularly valuable for rare defect types where labeled examples are scarce [290].

#### 5.3.3. Reinforcement Learning and Model Predictive Control

Reinforcement learning (RL) enables autonomous process control by learning optimal policies through interaction with the PBF environment. RL-based scan path optimization has demonstrated improved thermal uniformity and reduced residual stress through dynamic laser parameter adjustment, with geometry-agnostic controllers validated on Ti-6Al-4V and AISI 316L alloys [291]. Model predictive control (MPC) frameworks integrate RL with physics-based surrogate models, achieving precise melt pool temperature tracking that outperforms traditional PID control while maintaining real-time computational feasibility [292].

#### 5.3.4. Physics-Informed Neural Networks

Physics-informed learning represents a paradigm shift toward hybrid modeling, where governing physical laws are embedded directly into neural network architectures. Physics-informed neural networks (PINNs) encode partial differential equations (heat equation, Navier–Stokes) as physics-informed loss functions, achieving temperature field predictions with less than 7% deviation from experimental measurements while requiring significantly reduced computational time compared to finite element simulations [220]. PINNs demonstrate strong generalization across unseen laser scanning strategies and geometries [222].

#### 5.3.5. Integration with Digital Twin Frameworks

The integration of AI with DT frameworks enables predictive and adaptive control capabilities that transcend traditional process monitoring. ML models serve as fast surrogate representations within the DT architecture, replacing computationally expensive physics simulations for real-time inference while maintaining physical consistency through physics-informed training [204]. Bidirectional feedback loops facilitate closed-loop control, where DT predictions inform laser power adjustments, scan path modifications, or layer-wise parameter tuning to prevent defect formation, while in situ sensor data continuously refine model predictions through online calibration [293]. Hybrid physics–ML approaches combine the interpretability and extrapolation capability of physics-based models with the flexibility and speed of data-driven methods [230].

Domain adaptation techniques enhance DT reusability across different machines and sensor configurations, improving anomaly detection accuracy when transferring models between platforms, reducing the retraining burden for industrial deployment [244]. Uncertainty quantification through Bayesian frameworks provides confidence bounds on DT predictions, enabling risk-aware decision-making and robust process optimization under variable conditions [223].

#### 5.3.6. Validation and Scalability Considerations

Validation strategies for AI-enabled DTs include ex situ characterization (X-ray computed tomography, metallography) and in situ monitoring correlation, with model accuracy benchmarked against experimental thermal profiles, melt pool dimensions, and microstructural features [202]. Scalability challenges arise from the computational demands of high-fidelity physics models, addressed through surrogate modeling, reduced-order representations, and GPU acceleration that enable multi-layer simulations with practical latencies [294].

Table 13 provides a systematic comparison of AI and machine learning methods for PBF digital twin integration, evaluating application domains, physics integration strategies, sensor modalities, validation approaches, computational performance, transferability, and key limitations.

**Figure 10:**
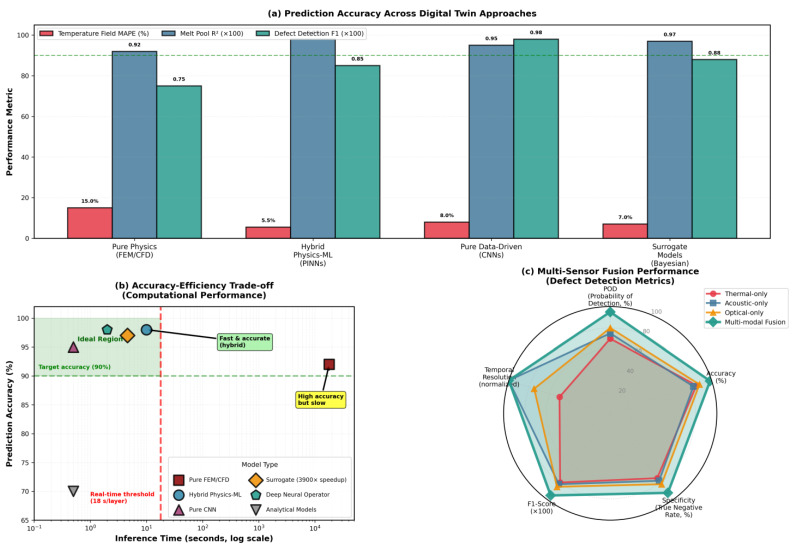
Performance of AI-enhanced digital twins: (**a**) accuracy of physics-based, PINN, data-driven, and hybrid models [210,212,267,276,277,5,194,213]; (**b**) accuracy–efficiency trade-offs showing 10–3900× speed-ups with ≥97% accuracy [5,194,210,213,214]; (**c**) multi-sensor fusion boosting defect detection to 98.5% accuracy and 95% POD, demonstrating real-time, closed-loop PBF capability [232,253,254,258,242,243]. Figure prepared using published data.

Figure 10 presents a performance overview of AI-enhanced digital twins, integrating accuracy, efficiency, and sensor fusion. Physics-based models achieve moderate accuracy, while physics-informed neural networks and hybrid surrogates markedly reduce errors and boost prediction fidelity. Computational trade-offs show hybrid and surrogate models, delivering up to 3900× faster performance than FEM/CFD with ≥97% accuracy, enabling real-time operation. Multi-sensor fusion further enhances defect detection to 98.5% accuracy and 95% POD by combining thermal, acoustic, and optical data. Together, these results highlight hybrid physics–ML digital twins with sensor fusion as the most effective framework for accurate, real-time control in industrial PBF systems.

### 5.4. Case Studies of PBF Digital Twin Implementation

Recent advances in digital twins (DTs) for industrial PBF highlight the effective integration of physics-based models and machine learning, enabling real-time process monitoring and control. Strengths include the development of hybrid digital twin architectures that combine data-driven and physics-based approaches, enhancing predictive accuracy and process optimization. However, challenges persist in scalability, data heterogeneity, and the integration of multi-source sensor data across diverse industrial contexts. Additionally, while many studies demonstrate promising capabilities in defect detection and process control, the generalizability and industrial deployment of these digital twins remain limited by computational demands and standardization issues. The synthesis highlights the need for modular validation frameworks and adaptable architectures to address evolving manufacturing complexities and ensure sustained digital twin fidelity.

An overview of digital twin developments for PBF is presented in Table 14.

## 6. Optimization Strategies in PBF Manufacturing

Reliable industrial deployment of powder bed fusion (PBF) increasingly depends on the integration of optimization frameworks with predictive quality assurance. Recent advances have demonstrated that embedding process physics into topology optimization enables designs that account for residual stresses, thermal distortion, and support minimization, although balancing computational fidelity with efficiency remains a challenge. At the same time, machine learning is transforming parameter selection, offering more accurate control of laser power, scan speed, and layer settings to enhance density, surface quality, and mechanical performance. Complementing these developments, predictive models that combine physics-based simulations with multi-sensor monitoring are emerging as powerful tools for defect detection and adaptive control. Collectively, these approaches mark a transition from empirical, trial-and-error strategies to integrated, data-driven frameworks capable of achieving first-time-right manufacturing in PBF.

**Figure 11:**
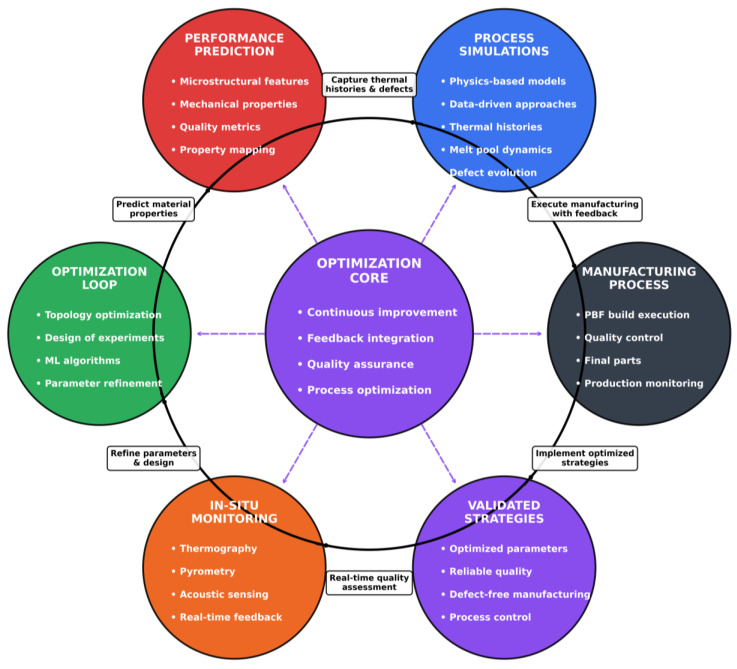
PBF process optimization. Figure conceptually synthesized by the authors from widely available literature and domain knowledge.

Figure 11 illustrates the iterative and continuous nature of optimization in PBF through a circular layout. At the center lies the optimization core, symbolizing the driving mechanism of constant improvement. Surrounding it are six interconnected components arranged in a logical sequence, each contributing to the process, a better understanding, and control. Feedback arrows radiating from the central core emphasize the integrated, bidirectional flow of information across all stages. Key annotations highlight the benefits of this framework, including adaptability, scalability, and enhanced process robustness, underscoring its role as a closed-loop pathway to reliable high-quality PBF manufacturing.

### 6.1. Topology Optimization with PBF Process Constraints

The studies on topology optimization in powder bed fusion additive manufacturing reveal significant advancements in integrating manufacturing constraints and process physics into design frameworks. Notably, the incorporation of thermal distortion, overhang control, and support structure optimization has been addressed with increasing sophistication. However, challenges remain in balancing computational efficiency with model fidelity, especially when simulating complex process-induced phenomena such as residual stresses and material heterogeneity. Furthermore, while multi-material and graded material considerations are emerging, their practical integration into topology optimization frameworks is still limited. The literature also highlights the growing role of artificial intelligence and machine learning in automating design for additive manufacturing, though these approaches require further validation. Overall, the field is progressing towards more holistic and manufacturability-aware optimization methods, yet gaps persist in experimental validation and scalability to industrial applications.

Table 15 highlights strategies and challenges in PBF-oriented topology optimization.

### 6.2. Machine Learning-Driven Process Parameter Optimization for PBF

Machine learning algorithms described in Section 5.3, including Bayesian optimization, deep neural networks, and reinforcement learning, are increasingly applied to optimize process parameters in powder bed fusion (PBF) additive manufacturing. Studies show significant improvements in build quality metrics such as surface roughness, porosity, and mechanical properties through intelligent parameter selection and real-time process monitoring.

Table 16 summarizes the application of ML and Bayesian optimization methods for PBF parameter optimization. A comparison of ML approaches for key PBF quality indicators is provided in Table 17.

### 6.3. Quality Assurance in PBF via Predictive Models

Predictive modeling for quality assurance applies the machine learning and sensor fusion methods detailed in Section 5.2 and Section 5.3 to defect detection and process control in PBF. While multi-modal sensor data integration and deep learning architectures enhance prediction accuracy and real-time monitoring capabilities, challenges persist in data acquisition, labeling, model generalizability across materials and process parameters, and computational efficiency for real-time applications. Robust, adaptable models that balance interpretability and performance while addressing practical industrial constraints remain a critical need.

## 7. Software Platforms and Tools for PBF Modeling

The computational ecosystem for powder bed fusion (PBF) modeling encompasses commercial finite element packages, open-source research codes, machine learning frameworks, and digital twin solutions. Tool selection depends on modeling objectives (process optimization, defect prediction, distortion analysis), required physics fidelity, computational resources, and licensing constraints. Commercial software offers mature implementations and technical support at a substantial cost, while open-source alternatives provide flexibility and transparency with steeper learning curves.

The multi-scale, multiphysics nature of PBF means no single platform comprehensively addresses powder spreading (DEM), laser–powder interaction (ray-tracing), melt pool dynamics (CFD), solidification microstructure (phase-field/cellular automata), and thermomechanical response (FEM) within a unified framework. Users typically employ multiple specialized tools coupled through custom scripts or APIs, introducing workflow complexity. This section reviews the principal software platforms that enable PBF modeling and digital twin development.

### 7.1. Commercial (e.g., ANSYS, Simufact) and Open-Source Tools

Simulation tools play a critical role in optimizing PBF processes by predicting distortions, residual stresses, and material properties. This section compares commercial and open-source tools, focusing on their technical capabilities, simulation accuracy, and computational efficiency, with specific examples of their applications in PBF.

ANSYS Additive is a commercial tool widely used for simulating PBF processes. It offers advanced capabilities such as predicting distortion, residual stress, and thermal deformation, as well as automatic support generation and scanning pattern optimization. The tool has been successfully applied to simulate the tension specimen of 17-4PH steel, demonstrating its ability to optimize process parameters and improve part quality [384,385]. ANSYS Additive uses finite element analysis to simulate the thermal history and mechanical behavior of parts during PBF. The tool can predict microstructural properties such as porosity and material anisotropy, which are critical for understanding the mechanical properties of the final part [385]. ANSYS Additive has been coupled with machine learning models to predict melt pool geometry and microstructure, significantly reducing simulation times compared to conventional numerical methods [385]. In a study on 17-4PH steel, ANSYS Additive was used to optimize process parameters, resulting in improved part quality and reduced production costs [384].

Simufact Additive is another commercial tool designed for simulating PBF processes. It is particularly useful for optimizing part orientation and predicting the influence of process parameters on part quality. The tool has been used to analyze the effect of weight factors on part orientation, demonstrating its ability to minimize support material volume and building risk [386]. Simufact Additive allows users to predict the optimal orientation of parts based on selected criteria, such as support material volume and building risk. The tool can simulate the residual stress and distortion caused by thermal gradients during the PBF process. Simufact Additive enables the assignment of weight factors to parameters, allowing users to prioritize specific criteria during optimization [386]. In a study on part orientation, Simufact Additive was used to compare three possible orientations of a part, demonstrating that orientation No. 1 achieved better mechanical properties compared to orientation No. 3 [386].

Other commercial tools, such as those from Autodesk and MSC, have also been benchmarked for PBF simulations. These tools were assessed for their ability to predict distortions and residual stresses in test geometries, with results validated using 3D scanning [387]. These tools can simulate the distortion caused by residual stresses during the PBF process. The accuracy of the simulations was validated by comparing the compensated parts with the original CAD design [387]. A case study involving Autodesk and MSC software demonstrated their effectiveness in predicting and compensating for distortions in PBF parts, highlighting their suitability for industrial applications [387].

OpenFOAM is a widely used open-source computational fluid dynamics (CFD) tool that has been adapted for PBF simulations. It has been modified to include the Marangoni effect, which is critical for accurately modeling heat transfer in the melt pool. The tool has been validated against experimental data, demonstrating its ability to simulate melt pool dynamics with high accuracy [388]. OpenFOAM has been enhanced to account for surface tension gradients, which are essential for modeling heat transfer in the melt pool. The tool can simulate multiple physical phenomena, including melting, solidification, and evaporation, making it suitable for complex PBF simulations. OpenFOAM’s parallel performance and scalability make it a viable option for large-scale PBF simulations [389]. OpenFOAM was used to simulate the influence of surface tension modeling on the mushy region in PBF, demonstrating improvements in the accuracy of melt pool dimensions and shape compared to experimental data [388].

AscentAM is an open-source simulation tool specifically developed for PBF processes. It uses a sequentially coupled thermomechanical approach to predict residual stresses and distortions. The tool has been validated for two benchmark geometries, demonstrating high accuracy in predicting distortion at different manufacturing states [390]. AscentAM simulates the thermal and mechanical behavior of parts during PBF, considering the physical relations of the process. The tool includes algorithms for part pre-deformation, enabling the optimization of part geometry before manufacturing. AscentAM’s predictions have been validated against experimental data, showing high accuracy in distortion prediction. AscentAM was used to simulate the distortion of a benchmark geometry, demonstrating significant reductions in form deviation through the application of optimization sub-modules [390].

Other open-source tools, such as those based on the deal.II library and particle-based methods have also been applied to PBF simulations. These tools focus on improving computational efficiency while maintaining high accuracy. For example, a highly parallelized and adaptive finite element method based on deal.II was used to analyze the accuracy and efficiency of thermomechanical simulations for Ti-6Al-4V parts [94]. These tools use adaptive mesh methods to reduce computational effort while maintaining accuracy. The use of parallel computing enables efficient simulation of large-scale PBF processes. Simplified material models and modeling assumptions are used to reduce computational costs without compromising accuracy [94]. A study using deal.II demonstrated that efficient simulations could achieve a maximum deviation of 8% in displacements and 3.5% in residual stresses compared to detailed simulations, while significantly reducing computational time [94].

Both commercial and open-source tools have demonstrated their effectiveness in simulating PBF processes, with each offering unique strengths. Commercial tools such as ANSYS Additive and Simufact Additive provide robust features for part optimization and residual stress prediction, while open-source tools like OpenFOAM and AscentAM offer cost-effective alternatives with high accuracy and scalability. The choice of tool depends on specific requirements, such as simulation accuracy, computational efficiency, and integration with experimental data.

### 7.2. Machine Learning Frameworks for PBF

Machine learning (ML) frameworks enable the development of data-driven surrogate models, process monitoring algorithms, and physics-informed neural networks (PINNs) for PBF applications [203,391,392]. General-purpose ML libraries provide building blocks (neural network layers, optimization algorithms, automatic differentiation) while PBF-specific implementations typically involve custom Python code, integrating these libraries with domain-specific data processing and physics constraints [367,393].

TensorFlow (Google) and PyTorch (Meta/Facebook) are widely used deep learning frameworks offering comprehensive neural network architectures (feedforward, convolutional, recurrent) and GPU acceleration capabilities. Researchers employ machine learning frameworks for developing defect detection algorithms from thermal images [270], LSTM-based temperature prediction [394], and surrogate models predicting melt pool dimensions or mechanical properties from process parameters [203,367].

Scikit-learn provides classical machine learning algorithms (support vector machines, random forests, Gaussian processes) and pre-processing utilities (feature scaling, dimensionality reduction) commonly used for PBF process parameter optimization, anomaly detection, and feature engineering [367,393,394]. Gaussian process regression (GPR) implementations enable Bayesian optimization approaches for experimental design and process parameter identification [394].

Physics-informed ML libraries embed governing equations into neural network training, improving generalization and data efficiency [391,392]. DeepXDE (Brown University) provides Python implementations of PINNs for solving PDEs, inverse problems, and data assimilation tasks [395]. These physics-informed approaches have been applied to predicting temperature distributions and thermal behavior in PBF processes [203,391].

Digital twin platforms provide infrastructure for real-time data ingestion, model orchestration, and visualization [263,396]. Cloud-based and open-source platforms are being explored for building digital representations of PBF systems, though domain-specific implementations require custom development for integrating these platforms with physics-based models and sensor interfaces [263].

### 7.3. Data Management and Visualization Tools

Effective data management and visualization tools are essential for handling the large volumes of sensor data, simulation results, and quality measurements generated during PBF processes. Multi-modal in situ monitoring datasets can reach hundreds of gigabytes per build. Managing and organizing these large data spaces require systematic frameworks following FAIR (Findable, Accessible, Interoperable, Reusable) principles to enable data integration and reuse [397].

In situ monitoring software integrates with machine sensors, providing real-time process visualization and anomaly detection [244,398]. Commercial solutions include Sigma Labs PrintRite3D for thermal monitoring [399], EOS EOSTATE for melt pool monitoring and exposure optimization on EOS machines [399], and various proprietary platforms from equipment manufacturers. Industry monitoring systems face challenges in data accessibility and interoperability, with data management and digital platform design being critical for qualification workflows [397,400]. Research applications often develop custom processing pipelines for sensor data acquisition, processing, and analysis, enabling flexibility for experimental investigations [244,398].

Data analysis in PBF research employs computational platforms for processing datasets spanning process parameters, sensor signals, and quality measurements. Python with scientific computing libraries (pandas for tabular data, numpy for numerical operations, scipy for scientific algorithms, matplotlib/seaborn for visualization) is widely used in research implementations due to open-source accessibility and extensive ecosystem support [244,398,401]. MATLAB remains common in academic research for signal processing and statistical analysis applications.

The visualization of three-dimensional simulation results and experimental data requires specialized software tools. Open-source visualization platforms such as ParaView and VisIt provide capabilities for large FEM/CFD datasets, though specific applications to PBF visualization workflows require custom development. Commercial FEM packages (ANSYS, Abaqus) include integrated post-processors with domain-specific visualization features for structural analysis results.

### 7.4. Integrated Platforms and Digital Twin Solutions

Emerging commercial solutions aim to provide end-to-end workflows spanning design, simulation, manufacturing, and quality assurance within unified platforms. Integration of the complete digital chain from design through manufacturing has been demonstrated through unified data structures and digital thread implementations [402,403,404].

Siemens NX has been employed for integrated CAD and additive manufacturing workflows including topology optimization and build preparation [402,405]. The platform enables design-for-additive-manufacturing (DfAM) workflows where simulation informs iterative design optimization [402,405]. However, for high-fidelity physics modeling, researchers often complement CAD-platform simulations with specialized finite element analysis codes [406].

Commercial CAD platforms offer additive manufacturing extensions with build orientation optimization, support generation, and distortion compensation capabilities, targeting accessibility for design engineers [407,408]. Physics fidelity and customization capabilities may be limited compared to dedicated FEM packages, leading researchers to employ specialized simulation tools for detailed analysis [406].

Research platforms typically involve custom integration of specialized tools through automated workflows and scripting interfaces [408,409]. Custom CAD-FEA integration approaches have been developed to enable DfAM-aware topology optimization and automated design workflows [409]. This approach provides flexibility and access to advanced methods but demands significant software engineering effort and development of custom data integration schemes [403,404].

## 8. Current Challenges and Future Directions in PBF Modeling

Significant progress in PBF modeling—from physics-based simulations to AI-augmented digital twins—has advanced understanding of process–structure–property relationships and enabled predictive process control in research settings. However, persistent challenges limit industrial deployment and certification for safety-critical applications. Table 18 systematically identifies these limitations across modeling approaches, while the following priorities outline pathways toward scalable, certifiable PBF manufacturing.

Table 18 synthesizes the principal challenges identified across modeling approaches reviewed in Section 3, Section 4, Section 5, Section 6, Section 7 and Section 8, organized by limitation category and supported by representative references.

**Figure 12:**
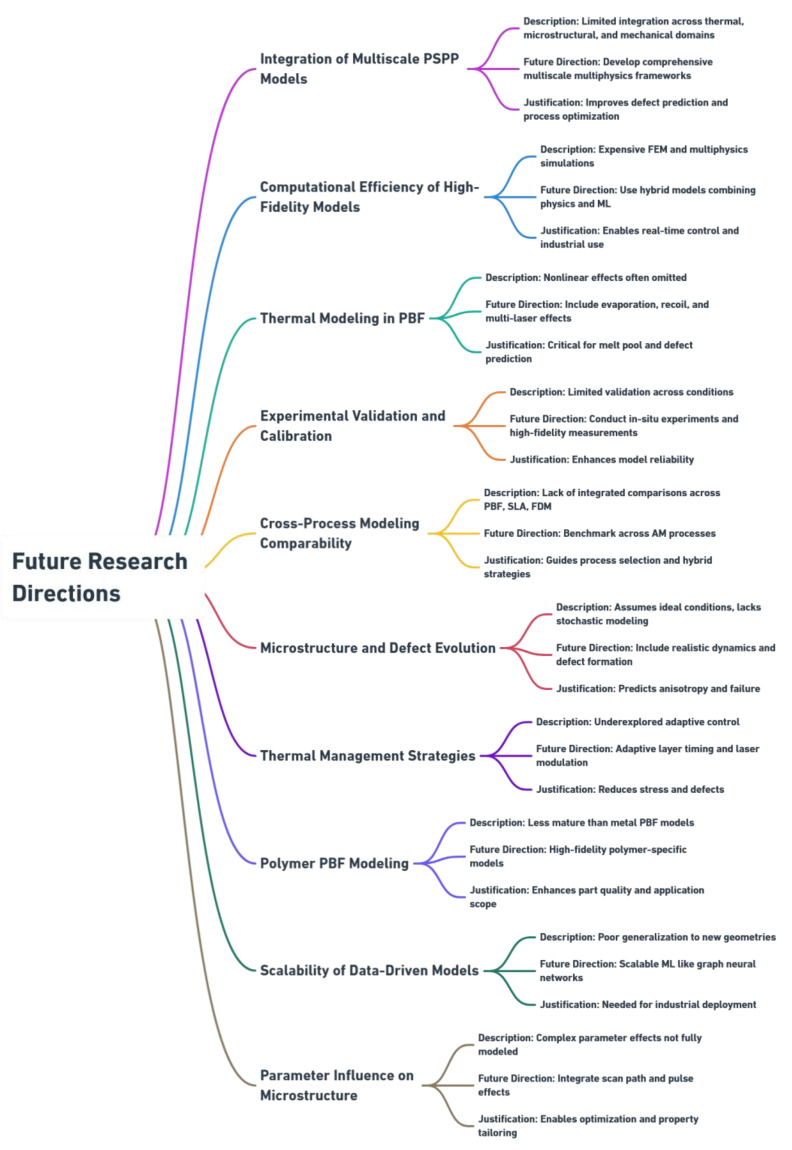
Overview of future research directions and priorities. Figure prepared using data and information reported in the cited publications.

The overarching challenge lies in achieving robust multi-scale and multiphysics coupling that seamlessly integrates thermal, mechanical, and microstructural phenomena while maintaining computational efficiency. Hybrid modeling frameworks blending physics-based simulations with machine learning surrogates show promise for balancing accuracy and speed. Figure 12 presents an overview of research directions addressing these challenges.

Achieving industrial viability requires immediate focus on validation infrastructure and computational scalability. Community-wide validation benchmarks with open-access experimental datasets—including thermal histories, defect distributions, microstructure maps, and mechanical properties—enable rigorous model verification across materials and machines. Open-source coupled simulation frameworks with GPU acceleration reduce computational barriers, while physics-informed machine learning surrogates achieve 100–1000× speed-ups, bridging high-fidelity predictions and real-time control. GPU-accelerated DEM codes enable part-scale powder spreading predictions, and integrated defect prediction algorithms combining multi-modal sensor fusion with validated ML models provide quantified detection accuracy suitable for quality assurance workflows.

Transitioning to certified manufacturing demands integrated digital twin platforms with closed-loop adaptive control validated on industrial-scale components. Real-time bidirectional synchronization enables layer-wise parameter adjustment and defect mitigation without post-process intervention. Multi-material and functionally graded modeling with validated interface predictions unlocks tailored property gradients and compositionally optimized structures. Uncertainty quantification frameworks providing statistically rigorous confidence bounds enable qualification-by-analysis pathways acceptable to regulatory agencies. Interoperable digital thread platforms with standardized data formats (FAIR compliance) facilitate seamless integration across design, manufacturing, and inspection systems. Explainable AI frameworks combining physics-informed architectures with interpretable feature attribution accelerate material qualification while maintaining regulatory transparency for safety-critical applications.

Realizing these priorities requires coordinated efforts across stakeholders. Academic institutions should prioritize open-source development and the publication of validation dataset. National laboratories should provide advanced characterization facilities, high-performance computing resources, and neutral platforms for pre-competitive consortia. The industry should contribute production process data, define acceptance criteria, and participate in standardized data format development through ASTM and ISO. Regulatory agencies should develop certification frameworks incorporating modeling evidence and establish guidelines for uncertainty quantification, supporting qualification-by-analysis pathways.

By systematically addressing these priorities, the PBF community can transform additive manufacturing into a robust, certifiable technology. The convergence of multiphysics modeling, artificial intelligence, in situ monitoring, and digital twins promises to unlock the full potential of powder bed fusion, enabling design freedoms and performance combinations unattainable through conventional manufacturing.

## 9. Summary

This review presents a comprehensive synthesis of modeling strategies for powder bed fusion (PBF), tracing their evolution from classical physics-based simulations to AI-augmented digital twins. By systematically evaluating advances across empirical models, numerical frameworks, multi-scale multiphysics approaches, hybrid physics–ML methods, and sensor-fusion-enabled digital twins, this work establishes how modeling has transformed understanding of process–structure–property relationships. Key contributions include the synthesis of scale-specific modeling approaches, critical assessment of coupled physics simulations, evaluation of microstructure prediction methods, analysis of in situ monitoring integration, examination of powder spreading dynamics, and review of digital twin architectures for real-time process control.

The integration of multiphysics modeling, artificial intelligence, in situ monitoring, and digital twins will enable powder bed fusion to evolve from empirical trial-and-error into a predictive, certifiable manufacturing technology, delivering design freedoms and performance characteristics beyond the reach of conventional fabrication methods.

## Figures and Tables

**Table 1:** Corpus composition.

Attribute	Distribution
Total Publications	408
Year Distribution	Pre-2020: 33 (8%)|2020: 31 (8%)|2021: 43 (11%)|2022: 62 (15%)|2023: 79 (19%)|2024: 111 (27%)|2025: 49 (12%)
Topics	Physics-based modeling (35%), ML/AI (28%), Digital twins (18%), Hybrid frameworks (12%), Monitoring/sensing (7%)
PBF Variants	LPBF: 87%|EB-PBF: 9%|Multi-laser/hybrid: 4%
Materials	Ti alloys: 32%|Ni superalloys: 24%|Stainless steels: 21%|Al alloys: 12%|Others: 11%
Methods	FEM: 38%|CFD: 18%|DEM: 14%|ML/NN: 22%|CA/Phase-field: 8%
Validation	Experimental: 68%|Benchmark: 22%|Sensitivity only: 10%

**Table 2:** Overview of the major PBF variants * [10,14,15,16,17,18].

Aspect	Selective Laser Sintering (SLS)	Selective Laser Melting (SLM)	Electron Beam Melting (EBM)	Multi Jet Fusion (MJF)
Energy Source	CO_2_ or fiber laser	High-power fiber laser	Electron beam	Thermal inkjet + infrared heating
Processing Environment	Nitrogen atmosphere	Inert gas (Ar/N_2_)	High vacuum (~10^−4^ mbar)	Nitrogen atmosphere
Working Principle	Selective sintering below the melting point	Complete melting of powder particles	Electron beam melting under vacuum	Chemical agent application + thermal fusion
Temperature Range	150–200 °C (powder bed)	Room temperature start	700–850 °C (preheated bed)	150–200 °C (powder bed)
Primary Materials	Polymers (PA12, PA11, TPU)	Metals (Ti, SS, Al, Co-Cr)	Metals (Ti alloys, Co-Cr, Inconel)	Polymers (PA12, PA11)
Typical Layer Thickness	50–200 μm	20–100 μm	50–200 μm	80–120 μm
Build Speed	Moderate (10–20 mm/h)	Slow (5–15 mm/h)	Fast (20–80 mm/h)	Fast (15–25 mm/h)
Part Density	85–95%	>99%	>99%	90–98%
Surface Finish	Ra 6–12 μm	Ra 5–15 μm	Ra 15–35 μm	Ra 3–8 μm
Dimensional Accuracy	±0.1–0.3 mm	±0.05–0.2 mm	±0.2–0.5 mm	±0.1–0.2 mm
Support Structures	Minimal (self-supporting)	Required for overhangs	Minimal (powder support)	Minimal (self-supporting)
Post-Processing	Powder removal, surface finishing	Support removal, heat treatment	Powder removal, machining	Powder removal, surface finishing

* (values represent typical ranges reported in the literature for optimized processing conditions; actual performance depends on specific machine configuration, material properties, part geometry, and processing parameters).

**Table 3:** Material properties relevant to PBF processing [4,18,19,20,21,23,24,25,26,27,28,30,31,32,44,47,48,49].

Alloy System	Thermal Conductivity (W/m·K)	Absorptivity	Solidification Range (°C)	Notable Microstructural Traits	Common Defects
Ti-6Al-4V	6.7–22 (temperature-dependent)	0.3–0.4 at laser wavelengths	1604–1660	Martensitic α’ phase, columnar β grains, epitaxial growth patterns	Porosity, lack of fusion, cracking, oxidation
AlSi10Mg	Low, anisotropic (5–15 typical)	0.1–0.3 (enhanced with nanoparticles)	577–660 (Al-Si eutectic)	Cellular dendritic structure, columnar-to-equiaxed transition	Lack of fusion, hot cracking, porosity
316L Stainless Steel	15–25 (varies with compaction)	0.35–0.45	1400–1450	Cellular austenitic structure, nanoscale oxide particles	Porosity, lack of fusion, residual stress
Inconel 625	10–15 (estimated)	0.3–0.4	1290–1350	Dendritic structure with Nb segregation, γ matrix	Solidification cracking, porosity, microsegregation
CoCrMo	14–17	0.4–0.5	1350–1450	HCP ε-martensite, FCC γ austenite	Porosity, phase transformation, and cracking
Maraging Steel (18Ni-300)	17–20	0.3–0.4	1413–1460	Martensitic laths, reverted austenite	Lack of fusion, thermal cracking
AlSi12	8–12 (anisotropic)	0.1–0.25	575–660	Fine cellular structure, Si precipitation	Hot cracking, porosity
Hastelloy X	9–12	0.35–0.4	1260–1355	Dendritic γ matrix with carbides	Solidification cracking, porosity

**Table 4:** Comparison of spreader types and their influence on powder layer quality in PBF.

Spreader Type	Packing Density	Surface Roughness	Speed Capability	Damage Risk	References
Rigid blade	0.58–0.62	4–7 μm	High (>300 mm/s)	Low	[59,64]
Flexible blade	0.60–0.64	3–5 μm	Medium (150–250)	Very low	[62]
Roller	0.60–0.63	3–6 μm	Medium (100–200)	Medium	[65,66]

**Table 5:** Influence of powder and process parameters on packing density and uniformity in PBF.

Parameter	Condition	Density	CV (%)	References
Morphology	Spherical (ψ > 0.95)	0.62–0.64	5–8	[73,74]
	Irregular (ψ < 0.85)	0.50–0.58	12–18	[72]
Speed	Optimal (100–200 mm/s)	0.60–0.63	6–10	[64]
	High (>300 mm/s)	0.52–0.58	15–20	[79]
Recoater	Flexible blade	0.60–0.64	5–8	[62]
	Roller (counter-rot.)	0.60–0.63	6–10	[66]
Temperature	Ambient (20 °C)	0.60–0.63	6–10	[70]
	Elevated (200 °C)	0.56–0.60	10–15	[70]
Gravity	Earth (1 g)	0.60–0.63	6–10	[68]
	Lunar (0.17 g)	0.52–0.58	12–18	[68]
Cohesion	Free-flow (H < 1.2)	0.62–0.64	5–8	[82]
	Cohesive (H > 1.3)	0.54–0.60	12–20	[71]

**Table 6:** Laser–powder interaction and energy absorption modeling approaches in powder bed fusion.

Model Type/Framework	Physics Captured	Resolution/Representation of Powder Geometry	Core Capabilities	Validation Strategy	Computational Cost/Efficiency	Key Limitations	Representative References
Analytical and Ray-Tracing Models	Multiple reflections, beam scattering, absorption, refraction, volume absorption within powder bed; laser energy distribution and attenuation; radiative transfer in particulate media	Accounts for particle size distribution, powder packing density, and layer thickness effects; can model random powder packing; some variants use idealized regular packing structures	High accuracy in predicting absorptivity variations due to powder bed structure; captures energy deposition patterns influenced by morphology; supports process parameter optimization for defect mitigation	Experimental absorptance data, profilometry, single-track experiments, thermal imaging, comparison with melt pool morphology	Medium: Monte Carlo and photon-particle tracking methods vary in efficiency; suitable for powder-scale simulations	Often assumes idealized powder bed structures, neglecting realistic morphology and surface roughness; limited representation of powder heterogeneity; requires coupling with thermal solvers for complete process modeling	[96,97,98]
Discrete Element Method (DEM) Coupled Optical–Thermal Models	Powder packing dynamics, particle-level morphology, surface roughness effects on absorptivity; laser–powder interaction, including multiple reflections; heat transfer through powder bed; powder degradation and recycled powder effects	Explicitly models realistic powder morphology, particle size distribution, shape effects (spherical, ellipsoids, irregular), packing density, and surface roughness; captures powder bed heterogeneity at the particle scale	Robust representation of absorptivity variations due to packing structure and morphology; validated against experimental powder bed characteristics and melt pool dimensions; strong capability for predicting powder bed quality effects on melt pool dynamics	Profilometry, microscopy, X-ray computed tomography (XCT) for powder bed validation, experimental melt pool metrics, single-track experiments, thermal profiles	Medium-to-high: GPU acceleration significantly reduces simulation time, enabling multi-layer and bulk-scale simulations; DEM frameworks computationally intensive for large domains	Computational expense limits scalability to full part simulations; multi-layer simulations remain challenging without GPU acceleration; requires extensive calibration for different materials; powder degradation effects underexplored	[61,81,99,100,101]
Continuum Models with Effective Medium Approximations (FEM/FVM)	Thermomechanical phenomena, heat conduction, phase change, residual stress, distortion; simplified representation of powder bed as homogeneous medium with effective properties; keyhole dynamics at threshold	Homogenizes powder bed characteristics using effective thermal conductivity and absorptivity; limited granularity on particle-level morphology; treats powder bed as a continuum	Moderate accuracy; effective medium approximations simplify powder-scale heterogeneity, potentially misrepresenting absorption and heat conduction; suitable for macro-scale thermal predictions; widely used for part-scale process control, distortion mitigation, and residual stress prediction	Experimental distortion data, residual stress measurements, melt pool metrics, thermal imaging, microstructure validation	Low to medium: highly efficient for part-scale simulations; enables multi-layer and full-part thermal–mechanical analysis	Oversimplifies powder bed characteristics; lacks resolution for local powder heterogeneities and melt pool instabilities; may overlook defect formation mechanisms dependent on powder morphology; limited representation of particle-scale physics	[102,103,104,105]
Particle-Based Methods (SPH, LBM) for Radiation–Heat Coupling	Detailed melt pool fluid dynamics, phase change, melting mode transitions, recoil pressure, Marangoni convection, evaporation, heat transfer and solidification, powder–melt interactions, powder wetting influence on morphology	Models powder thermal conductivity and particle-scale interactions; captures fluid flow around powder particles; SPH and LBM handle complex free surface dynamics and powder wetting	High fidelity in capturing melt pool dynamics and phase transitions; accurate representation of melting behavior and keyhole formation; validated against experimental melt pool geometry; effective for detailed process understanding of melt pool instabilities	X-ray analyses, high-speed imaging, melt pool geometry validation, experimental track morphology, thin wall morphology, in situ monitoring	High: computationally intensive, limiting scalability to particle or mesoscale domains; restricted to single-track or small domain simulations	Computationally prohibitive for multi-layer or full-part simulations; limited industrial deployment due to computational demands; often excludes full vapor dynamics; scalability challenges for industrial applications	[84,106,107,108]
Hybrid Multiphysics Models (Ray-Tracing + FEM/CFD + Microstructure)	Comprehensive multiphysics: optical absorption via ray-tracing combined with thermal–fluid dynamics (phase change, recoil pressure, Marangoni effects), solidification, microstructure evolution (cellular automata, phase-field); bubble generation and migration	Integrates detailed powder packing (via DEM or explicit morphology) with continuum thermal analysis; captures powder bed heterogeneity and its influence on energy absorption and heat transfer; spatial beam shaping effects	High accuracy combining detailed optical physics with thermal–fluid predictions; validated against melt pool dimensions, temperature fields, and microstructure; supports digital twin development; enables prediction of defects (porosity, keyholing) and microstructure	XCT, thermal imaging, pyrometry, in situ X-ray monitoring, melt pool metrics, experimental microstructure data, single-track and multi-track validation	Medium: balances fidelity and efficiency through modular coupling; GPU acceleration improves feasibility for multi-layer simulations	Coupling complexity increases model development effort; trade-offs between resolution and computational feasibility; requires extensive experimental validation; integration of vapor dynamics and spatter remains incomplete; standardized validation protocols lacking	[109,110,111,112]

**Table 7:** Melt pool modeling approaches in powder bed fusion.

Model Type/Category	Physics Captured	Core Capabilities	Validation Methods	Computational Cost/Efficiency	Limitations/Challenges	Representative References
Analytical and Semi-Analytical Models	Simplified heat conduction; process-dependent laser absorptivity; heat source modeling (Rosenthal, Goldak); temperature-dependent properties in extended versions; conduction-dominant heat transfer	Closed-form solutions for thermal field prediction; rapid melt pool geometry estimation (width, depth); process parameter mapping; printability window identification; suitable for rapid process parameter screening	Ex situ validation with experimental melt pool dimensions; thermal profile measurements; validated with ~7–8% discrepancy for melt pool dimensions	Very high efficiency: computationally inexpensive (<200 s in some cases); orders of magnitude faster than numerical simulations; suitable for real-time applications	Neglects fluid flow, Marangoni convection, recoil pressure, and keyhole dynamics; assumes conduction-dominant heat transfer; limited accuracy for transient phenomena and keyhole regimes; requires empirical calibration for quantitative precision; lacks detailed defect prediction capability	[126,127,128]
Continuum Numerical Models (FEM/FVM for Thermal–Fluid Coupling)	Comprehensive heat transfer (conduction, convection, radiation); fluid flow with Marangoni convection and recoil pressure; phase change and solidification; surface tension; vaporization effects; temperature-dependent material properties	Detailed thermal history prediction; melt pool morphology and dimensions; thermal gradients and cooling rates; solidification behavior; supports defect prediction including porosity and lack of fusion; multi-layer and multi-track simulations	Ex situ validation with experimental temperature profiles, melt pool geometry, thermal history; high-speed imaging correlation; validated with good agreement on temperature gradients and melt pool morphology	Moderate to high cost: computationally intensive for multi-layer or multi-track simulations; adaptive meshing and parallelization improve efficiency; ~4.4× faster than traditional FEM in optimized FVM implementations	Mesh-based methods face challenges with violent interface dynamics and complex free surface flows; require remeshing or interface tracking algorithms; high computational demands limit scalability to large-scale simulations; simplified powder bed morphology in some implementations	[104,129,130,131,132]
High-Fidelity CFD with Multiphysics Coupling	Comprehensive thermal–fluid dynamics; Marangoni convection; recoil pressure; surface tension; evaporation and vaporization; phase change and solidification; powder–laser interaction; keyhole formation, vapor depression, and oscillations; sulfur-induced flow transitions	High-accuracy prediction of melt pool size, shape, temperature distribution, and fluid flow patterns; captures conduction and keyhole modes; defect formation mechanisms including porosity (lack of fusion, keyhole pores), balling, spatter, inter-track voids, and bubble dynamics; fluid instabilities and melting-solidification characteristics	Ex situ validation with experimental melt pool dimensions, temperature measurements, high-speed imaging; in situ monitoring data correlation; X-ray imaging for keyhole dynamics; validated against synchrotron data	Moderate-to-high demand: multiphysics coupling increases complexity and computational cost; computationally intensive, limiting scalability for multi-layer and complex geometries	Computationally intensive; limited scalability for industrial-scale multi-layer simulations; sensitivity to numerical parameters; requires accurate boundary conditions and material properties; complex powder bed morphology often simplified; integration challenges across scales	[116,117,133,134,135]
Mesh-Free and Particle-Based Methods (SPH, LBM, DEM)	SPH: fluid flow with complex free surface dynamics, Marangoni convection, recoil pressure, surface tension, evaporation, thermo-capillary flow, phase transitions, melting mode transitions; LBM: fluid flow, heat transfer, phase change, keyhole oscillations, vapor capillary evolution; DEM: powder packing, spreading, granular dynamics, particle-level interactions	SPH: detailed melt pool dynamics, captures powder behavior and melting, fluid flow with complex interfaces, superior handling of violent interface dynamics, balling defects, keyhole formation; LBM: melt pool fluid dynamics, keyhole oscillation dynamics; DEM: powder bed characterization, powder spreading simulation, powder wetting effects on thin wall morphology	Ex situ validation with experimental melt pool shapes, surface temperatures, defect mechanisms (balling), thin wall morphology; high-speed imaging and synchrotron data correlation; validated qualitatively and quantitatively with experiments on melting mode transition, vapor depression geometry	Moderate-to-high cost: SPH moderate with incompressible schemes and optimized solvers; computationally expensive for large-scale simulations; DEM moderate for powder packing; LBM high for detailed fluid dynamics; both limit scalability	SPH: careful calibration of smoothing lengths and kernel functions required; less mature integration with solid mechanics; particle discretization simplifications; LBM: complexity in multi-phase flows; DEM: simplifications in particle interactions; limited integration with macro-scale models; scalability constraints for industrial applications	[84,91,107,136,137,138]
Multi-Scale Coupled Models (Phase-Field, CA, Physics-Informed ML)	Phase-Field/CA: coupled thermal–fluid dynamics with rapid solidification kinetics, grain growth mechanisms, solid/liquid phase transitions, Marangoni convection, recoil pressure, microstructure evolution; ML/PINN: implicitly models temperature fields, melt pool boundaries, thermal gradients, physics-guided architectures encode process parameter dependencies	Phase-Field/CA: links melt pool dynamics to microstructure outcomes (grain size, morphology), predicts porosity and grain structure, supports process–structure–property relationships; ML/PINN: rapid prediction of 3D thermal fields and melt pool geometry (2–3% error), strong generalization across process parameters, near-instantaneous inference after training	Phase-Field/CA: ex situ validation with microstructure characterization (grain size, morphology), porosity measurements, melt pool dimensions; ML/PINN: trained on high-fidelity CFD/FEM simulation data and experimental datasets; validated against experimental melt pool dimensions and thermal measurements	Phase-Field/CA: high cost due to multiphysics and multi-scale coupling; computationally intensive, limiting domain size; ML/PINN: very high efficiency for inference after training; orders of magnitude faster than physics-based simulations	Phase-Field/CA: computationally intensive; limited to small domains or simplified geometries; complex coupling strategies; requires extensive calibration; scalability challenges for industrial-scale simulations; ML/PINN: requires extensive high-quality training data; limited physical interpretability; extrapolation beyond trained parameter ranges unreliable; generalizability depends on training data diversity	[110,111,139,140,141]

**Table 8:** Influence of key parameters on residual stress and distortion in PBF.

Parameter	Condition	Effect on Peak Residual Stress	Effect on Distortion	Mechanism	References
Preheating Temperature	Ambient (20 °C)	Baseline (500–700 MPa for Ti-6Al-4V)	Baseline (0.3–0.5 mm for 50 mm parts)	High thermal gradients, limited stress relaxation	[156,157]
	Elevated (200–300 °C)	Reduced by 40–60%	Reduced by 50–70%	Lower thermal gradients, enhanced plastic relaxation	[156,166,168]
Laser Absorptivity	Low (A = 0.30–0.35)	Reduced by 15–25%	Reduced by 10–20%, risk of lack of fusion	Reduced energy coupling, smaller melt pool, lower thermal gradients	[171,172]
	High (A = 0.55–0.70)	Increased by 15–25%	Increased by 10–20%	Enhanced energy deposition, larger melt pool, higher thermal gradients	[156,173]
Laser Power	Low (150–180 W)	Moderate (300–450 MPa)	Moderate (0.2–0.3 mm), fusion quality risk	Reduced melt pool size, lower peak temperatures	[156,168]
	High (230–280 W)	Elevated (550–750 MPa)	Elevated (0.4–0.6 mm)	Enlarged melt pool, increased thermal gradients and plastic strain	[156,174]
Scan Speed	Low (600–800 mm/s)	Elevated (500–700 MPa)	Elevated (0.4–0.5 mm)	High energy density, extended melt pool, cumulative heating	[168,174]
	High (1000–1400 mm/s)	Reduced (350–500 MPa)	Reduced (0.2–0.3 mm), fusion quality risk	Low energy density, reduced thermal interaction	[156,174]
Hatch Spacing	Small (0.08–0.10 mm)	Moderate to elevated (450–600 MPa)	Moderate (0.3–0.4 mm)	High track overlap, re-melting, cumulative heating	[168,172]
	Large (0.12–0.15 mm)	Reduced (350–500 MPa), lack-of-fusion risk	Reduced (0.2–0.3 mm)	Low track overlap, reduced thermal interaction	[172,174]
Laser Operation Mode	Continuous Wave (CW)	Baseline (500–700 MPa)	Baseline (0.3–0.5 mm)	Steady-state thermal gradients, continuous melting	[156,171]
	Pulsed (optimized)	Reduced by 20–40% (300–500 MPa)	Reduced by 25–45% (0.15–0.3 mm)	Intermittent heating, stress relaxation during pulse-off, reduced peak gradients	[169,171]
Scanning Strategy	Unidirectional	Anisotropic stress (300–600 MPa range)	Anisotropic (0.2–0.5 mm directional)	Directional thermal gradients, accumulated strain along scan	[167,175]
	Island/Checkerboard	Reduced, more isotropic (350–500 MPa)	Reduced by 30–50% (0.15–0.3 mm)	Localized heating, reduced global thermal gradients, stress compartmentalization	[176,177]

**Table 9:** Comparative evaluation of modeling approaches in PBF.

Modeling Approach	Physics Captured	Core Capabilities	Validation Strategies	Computational Efficiency	Scalability	Transferability	Industrial Applicability	Key Limitations	References
Empirical and Semi-Empirical Models (Regression-Based Correlations)	Effective thermal behavior captured through statistical correlations; limited explicit physics representation	Rapid prediction of melt pool dimensions, temperature histories, and thermal characteristics; process parameter mapping; printability window identification	Calibrated with experimental data; validated against melt pool geometry and thermal measurements	Very high computational efficiency; low computational cost, enabling rapid predictions suitable for process mapping and control	Limited scalability to complex geometries; primarily suited for part-scale and process-scale simulations	Limited transferability; heavily dependent on experimental calibration; constrained to tested materials and process conditions	Suitable for rapid process optimization, preliminary design space exploration, and printability assessments; enables quick process parameter studies	Lack mechanistic insight; oversimplify complex thermal phenomena; neglect fluid flow and phase changes; require extensive calibration; limited generalizability beyond specific experimental conditions	[127,189,190,191]
Analytical Models (Rosenthal-Type Heat Conduction, Moving Point Heat Source)	Dominant heat conduction with simplified boundary conditions; temperature-dependent properties in extended versions; conduction, convection, radiation, and melting losses in advanced formulations	Closed-form or semi-closed-form solutions for thermal field prediction; melt pool geometry estimation; rapid thermal history predictions; residual stress estimations; process window identification	Ex situ validation against experimental thermal profiles and melt pool dimensions; validated with thermographic measurements; sensitivity analyses on assumptions	Highly efficient; orders of magnitude faster than full numerical simulations; suitable for rapid predictions (<200 s in some cases)	Part-scale thermal modeling; applicable across various scanning strategies; limited to simplified geometries and boundary conditions	Moderate transferability; requires empirical calibration for quantitative precision; assumptions limit applicability to new materials without adjustment	Facilitates rapid process parameter mapping; supports printability assessments and process control strategies; useful for initial design and optimization	Rely on simplifying assumptions (steady-state, linear heat conduction, idealized heat sources); neglect fluid flow, keyhole effects, and complex melt pool dynamics; limited accuracy for transient and multiphysics phenomena; require empirical corrections for nonlinear effects	[119,128,192,193]
Semi-Analytical Models with Nonlinear Corrections	Heat conduction with temperature-dependent material properties; incorporates nonlinear effects and phase change; includes convection and radiation boundary conditions	Enhanced thermal field prediction with improved accuracy over linear analytical models; residual stress and deformation prediction; rapid part-scale thermal modeling	Ex situ validation with experimental data; ~90% accuracy validated in some implementations; sensitivity analyses on material properties	Very efficient compared to full numerical models; minimal computational overhead vs. linear analytical models; enables rapid iterative design	Part-scale thermal and thermomechanical modeling; applicable to various scanning patterns and geometries	Improved transferability over pure analytical models; still requires empirical calibration; better generalization with nonlinear corrections	Supports efficient process optimization and defect prediction; balances accuracy and speed for industrial applications; useful for process control	Linear base model assumptions with empirical calibration; still neglects detailed fluid flow and complex melt pool hydrodynamics; limited to conduction-dominant scenarios	[194,195,196]
Finite Element Method (FEM) for Thermomechanical Modeling	Comprehensive heat transfer (conduction, convection, radiation); thermomechanical coupling; residual stress and distortion; phase transformations; temperature-dependent material properties	Detailed thermal history prediction; residual stress and deformation analysis; part-scale distortion prediction; multi-layer simulations; microstructure evolution coupling	Ex situ validation with experimental measurements; validated against distortion, residual stress measurements; ~8% displacement and ~3.5% stress error in optimized implementations	Moderate-to-high computational cost; computationally intensive for multi-layer or part-scale simulations; adaptive remeshing and parallelization improve efficiency	Powder to part scale; scalable with adaptive strategies and model simplifications; suitable for multi-layer and complex geometries	Good transferability across materials with proper calibration; material property databases support cross-material application	Industry standard for detailed thermal and mechanical analysis; supports design optimization, distortion mitigation, and qualification; enables digital twin integration	High computational demands limit real-time applicability; requires extensive material property data; mesh generation complexity; simplified powder behavior in some implementations; linearized material models reduce accuracy	[2,94,197,198]
Finite Volume Method (FVM) for Thermal Analysis	Heat conduction dominant; includes convection and radiation; temperature-dependent properties; phase change effects	Thermal field prediction; temperature history calculation; melt pool thermal analysis; efficient heat transfer simulations	Ex situ validation; good accuracy vs. FEM benchmarks; validated against experimental thermal profiles	Higher computational efficiency than FEM (~4.4× faster reported); moderate computational cost with improved solver efficiency	Part-scale thermal analysis; applicable to large domains with efficient discretization	Good transferability with proper boundary condition specification; similar to FEM in cross-material applicability	Suitable for rapid thermal analysis in industrial settings; supports process optimization with reduced computational burden vs. FEM	Simplified fluid flow effects; less mature than FEM for multiphysics coupling; limited representation of complex melt pool dynamics; requires proper boundary condition specification	[103,199]
Smoothed Particle Hydrodynamics (SPH) and Particle-Based Methods	Comprehensive fluid flow and heat transfer; free surface tracking; phase change and solidification; powder–laser interaction; granular flow dynamics; melt pool hydrodynamics	Detailed melt pool dynamics modeling; captures powder behavior and melting; fluid flow with complex interfaces; powder spreading simulation; multi-phase interactions	Ex situ validation with experimental melt pool morphology; validated against synchrotron imaging; benchmarked with experiments on welding and AM	Computationally expensive; high computational cost limits large-scale applications; moderate-to-high cost depending on particle resolution	Powder-to-melt pool scale; mesoscale to macro-scale applicability; limited scalability to full part-scale due to computational demands	Moderate transferability; flexible for different materials and process conditions; assumptions in particle interactions affect transferability	Effective for detailed melt pool and powder dynamics research; supports fundamental process understanding; useful for phenomena not captured by continuum methods	Computationally intensive; complex boundary and interface assumptions; less mature than mesh-based methods; particle discretization simplifications; limited to small-scale or mesoscale simulations in practice	[91,106,184]
Lattice Boltzmann Method (LBM) and Discrete Element Method (DEM)	LBM: fluid flow, heat transfer, phase change at mesoscale; DEM: powder packing, spreading, granular dynamics, particle-level interactions	LBM: melt pool fluid dynamics and thermal modeling; DEM: powder bed characterization, powder spreading simulation, powder–laser interaction modeling	Ex situ validation; DEM validated against powder bed characteristics; LBM validated for fluid flow and thermal fields	Computationally expensive; DEM for powder packing moderate cost; LBM for fluid dynamics, high cost; both limit scalability	Powder scale to mesoscale; DEM effective for powder bed modeling; LBM for melt pool scale; limited to small domains	Transferable across powder materials for DEM; LBM requires calibration for different fluids and materials	DEM useful for powder bed quality assessment and spreading optimization; LBM for detailed melt pool physics research; supports process understanding	High computational cost limits practical application; DEM simplifications in particle interactions; LBM complexity in multi-phase flows; limited integration with macro-scale models; scalability constraints	[103,200]
Multi-Scale and Multiphysics Integrated Frameworks	Coupled thermal, fluid, mechanical, and microstructural physics; heat transfer, mass transfer, phase transformations; powder dynamics to part-scale phenomena	Comprehensive process simulation across scales; links powder behavior to melt pool dynamics to part-scale properties; microstructure evolution prediction; defect formation analysis	Multi-scale validation with experimental data at different scales; validated against thermal, mechanical, and microstructural measurements	Computationally intensive; high computational cost due to multiphysics coupling; efficiency improvements via adaptive strategies and scale separation	Multi-scale from powder to part scale; framework supports integration across scales but increases complexity	Enhanced transferability through physics-based foundations; multi-scale coupling improves generalization but requires extensive calibration	Supports comprehensive process understanding and optimization; enables process–structure–property linkage; useful for digital twin development and advanced process control	Complexity in coupling strategies; high computational demands limit practical deployment; simplifications in coupling physics; data requirements for validation; integration challenges across scales; model calibration complexity	[8,112,201,202,203]

**Table 10:** Hybrid physics–data-driven modeling approaches.

Model Category	Application Domain	Physics Integration Strategy	Validation Method	Computational Efficiency	Transferability/Scalability	Key Limitations	References
Physics-Informed Neural Networks (PINNs)	Temperature field and melt pool geometry prediction; thermal history modeling; parameter identification; real-time process monitoring and adaptive control	Strong coupling: governing PDEs (heat equation, Navier–Stokes) embedded as physics-informed loss functions; conservation laws integrated into network training; dynamic weight updates with physics-informed constraints; ontology-integrated frameworks	Ex situ validation with FE simulations and experimental data; in situ validation with real-time anomaly prognosis; validated across scanning speeds, process parameters, and 3D benchmark problems; errors below 3% for 2D temperature fields	High efficiency: computational time significantly reduced vs. pure FEM/CFD (MAPE 2.8%, R^2^ 0.936); efficient with limited and sparse data; enables real-time monitoring and adaptation	Generalizes to unseen laser scanning strategies and geometries; transferable to different builds without retraining; demonstrated on NIST benchmark parts; adapts to new materials and parameters	Requires partial experimental data; accuracy depends on physics model fidelity; most models focus on 2D or single-track simulations; integration of multi-sensor data streams and handling noisy data remain challenges; requires valid digital twin	[140,218,219,220,221,222]
Deep Neural Operators and Physics-Based Surrogate Models for Digital Twins	High-fidelity melt pool state prediction; closed-loop feedback control; temperature and defect prediction with online calibration; digital twin integration for adaptive manufacturing	Hybrid: physics-based surrogate models combined with deep neural operators (Fourier Neural Operators); offline fine-tuning with physics simulations; incorporates uncertainty quantification and Bayesian calibration	Closed-loop feedback control with online updates; ex situ validation on experimental datasets; supports adaptive calibration; validated with multi-fidelity FE and ML calibration	High efficiency: orders of magnitude faster than traditional FE simulations; supports near-real-time inference; fast surrogate enables efficient closed-loop control	Adaptable via offline fine-tuning and calibration; incorporates uncertainty quantification for evolving conditions; supports layer-by-layer parameter adjustment	High computational cost for initial high-fidelity simulations; complexity of model integration; dependence on synthetic or simulated data may not capture all experimental variability; calibration against limited experimental datasets remains bottleneck	[5,7,223,224]
Hybrid CFD/FEM–ML Surrogate Models	Melt pool width and geometry prediction; defect formation prediction; process parameter optimization; printability mapping and qualification acceleration	Modular coupling: CFD/FEM simulations provide training data for ML surrogates (SVM, U-Net, ensemble methods); integrates experimental and simulation data; data-driven methods augment physics-based predictions; scientific ML with physically intuitive features	Ex situ validation integrating experimental and simulation data; validated on Ti-6Al-4V, AlSi10Mg, and multiple alloys; combines simulation and empirical data; R^2^ > 0.98 for melt pool geometry	High efficiency: faster than high-fidelity CFD/FEM; accelerates qualification of LPBF parameters; reduces error norms by up to 75%; relative mean absolute error ~6.77%	Effective under sparse data with multiple chemistries; considers powder bed thickness and preheating effects; integrates simulation and experimental data; outperforms black-box models	Not explicitly real-time but supports process insight; limited to specific parameter sets or small geometries; dependence on simulation quality for training data; transferability across machines and materials requires further validation	[204,225,226,227]
Physics-Guided Generative Models and Hybrid Neural Networks	Melt pool behavior prediction; defect identification (lack of fusion, porosity, keyhole); built quality and defect type prediction; anomaly detection	Embedded physics: generative adversarial networks (GANs) guided by physics; hybrid neural networks fusing thermal images and simulated melt pool images; mechanistic model integration with synchrotron X-ray data	Ex situ validation with experimental data; 97.25% defect identification accuracy; multi-classification framework for quality prediction; experimentally validated across multiple regimes	High efficiency: accelerated prediction vs. traditional methods; reduced CFD computational cost; efficient multi-classification framework; temperature metrics with 5–15% uncertainty	Validated experimentally; combines physical and data features for robustness; explores parameter influence on quality; robust under sparse data; tested across regimes	Image prediction quality prioritized over exact quantitative agreement in some cases; limited validation on complex geometries; reliance on synthetic data for training; accuracy depends on quality of physics simulations	[228,229,230,231]
Physics-Based Thermal Models with ML for Microstructure Evolution	Microstructure evolution prediction (primary dendritic arm spacing, grain growth, melt pool depth); scan path optimization for microstructure control; process–structure–property linkage	Physics-based thermal model inputs to ML (SVM, U-Net surrogates); combines phase-field modeling with ML; mechanistic modeling integrated with ML; incorporates recoil pressure and fluid flow effects	Ex situ validation; validated on multi-layer and multi-track cases; experimentally validated microstructure predictions; RMSE ~0.012 mm for melt pool depth, 110 nm PDAS	High efficiency: rapid part-level thermal model with ML prediction; reduces computational time by ~1000× vs. phase-field simulations; enables intelligent process optimization	Transferable to different builds without retraining; demonstrated on NIST benchmark parts; geometry-agnostic capabilities; applied to Inconel 625 and molybdenum materials	Scalability to full parts or complex geometries limited; high computational cost when applied to full parts; most models focus on single-track or few-layer scenarios; limited validation on complex multi-layer builds	[203,232,233]

**Table 11:** In situ monitoring and data fusion approaches for improved understanding of the PBF process.

Monitoring Approach	Sensor Types and Data Characteristics	Validation Level	Computational Performance	Transferability	Industrial Readiness	Best Use Cases and Rationale
Thermal/IR Imaging for Melt Pool and Layer Monitoring	High-speed NIR/LWIR cameras, imaging spectrometers, mid-IR collectors; spatial resolution down to 50 µm (ADM optic); layer-wise thermal maps [234,235,236].	Validated via registration to XCT and optical microscopy for pore morphology/distribution across multiple geometries [235,236,237].	High frame rates (specific rates vary); temperature–emissivity separation (TES) achieves ±28 K accuracy over 1000 K range [234]; many systems require offline processing for mapping [4,11].	Demonstrated on Ti, 316L, Ni superalloys, diverse geometries; cross-machine generalization limited to reported testbeds [235,236,238].	TRL 5–6; ADM achieved micro-CT correlation to 4.3 µm pores on testbed; not yet turnkey industrial product [235].	Best for layer-wise anomaly localization, thermal history tracking, correlating surface thermal signatures to XCT porosity maps, as spatial resolution enables precise defect localization when coupled with rigorous registration [234,235,236].
Photodiode-Based Monitoring	On-axis photodetectors, ratiometric bichromatic sensors measuring integrated melt-pool emission; calibrated against tungsten lamp and blackbody [239,240].	Calibration studies and repeatability checks on testbeds; signals correlated to density patterns and edge effects [239,240].	High temporal bandwidth; sampling rates/inference latencies not comprehensively reported [239].	Used across different machine optics with normalization strategies; broad cross-machine transfer claims limited [239,240].	TRL 7–8; widely integrated on commercial systems; NIST calibration guidance exists [240].	Best for fast melt-pool event detection, global trend monitoring, feedstock/optics health checks, as single-point high-bandwidth signals enable rapid anomaly detection without spatial imaging overhead [239,240].
Multi-Sensor Fusion (Visible/NIR/SWIR/LWIR/Acoustic)	Fusion of visible, NIR, SWIR, LWIR, optical tomography, acoustic emission, back-reflection, scan metadata in voxelized footprints [241,242,243,244].	Strong XCT validation; voxel-by-voxel binary classification: 98.5% accuracy; POD for 200–1000 µm flaws with a 90/95 metrics [242,243,244].	Neural networks and variational autoencoders; low-latency designs targeted but detailed metrics sparse; human-in-the-loop annotation improves POD [243,244].	Demonstrated on testbeds and industrial components; multi-laser cases explored; generalization often requires re-annotation/retraining [235,243,244].	TRL 5–6; INDE framework shows production-scale engineering with POD reporting consistent with NDE practice [243,244].	Best for detecting multiple defect types (lack-of-fusion, keyhole, subsurface porosity) and producing probabilistic POD curves for qualification, as multi-modal data captures complementary physics; 98.5% classification accuracy and POD metrics support certification workflows [242,243,244].
Machine Learning for Defect Detection and Classification	Deep CNNs, U-Net variants, CNN + LSTM hybrids, variational autoencoders on single/fused sensor data [242,245,246].	Ground truth: XCT; reported accuracies 97.86–98.5% for defect/regime classification; voxel classification via cross-validation [242,245,246].	Rasterized layer images or voxelized footprints; 0.5–4 ms regime detection targeted; many pipelines trained offline [246].	Demonstrated across geometries/materials within study scope; applied to industrial geometries with retraining [243,245].	TRL 4–6; high-performance lab/testbed demonstrations; some integrated into analytics platforms; full factory closed-loop limited [243,244].	Best for supervised in situ detection where extensive XCT ground truth is available; producing POD/PDFA metrics for qualification, as high classification metrics (≥97.86%) when trained on rigorous XCT-labeled data [242,245,246].
Data Registration Techniques (In Situ to XCT)	Image-to-volume registration, adaptive volume adjustment, fiducials, deformation modeling to align thermography/optical tomography to XCT [237,247].	Necessary and effective for correlating in situ signals to XCT; adaptive methods improve alignment accuracy and reduce false positives/negatives [237,247].	Typically offline and computationally intensive; real-time registration latencies not reported for full volumes [237,247].	Applied across multiple specimen shapes (cylinders, complex geometries); fiducials and deformation models improve cross-part mappings [237,247].	TRL 3–5; critical enabling step for ML training and POD estimation; research-grade, not embedded in real-time factory systems [237,247].	Best for producing trustworthy ground truth alignment between in situ signals and XCT for ML training and POD estimation, as registration accuracy directly impacts ML model reliability and defect localization precision [237,247].
Temperature Calibration and Emissivity Measurement	Multi-wavelength pyrometry, temperature–emissivity separation (TES), pixelwise camera calibration, tungsten lamp reference [234,238,239,248,249].	TES: ±28 K retrieval accuracy over 1000 K range; pixelwise calibration: 500–1500 K; multi-wavelength studies show 20–300% emissivity variation across alloys/phases [234,238,248,249].	Requires spectrally resolved sensors and careful optics; computational cost of TES/pixelwise fitting reported; real-time absolute temperature mapping challenging [234,249].	Emissivity varies by alloy and process stage; multi-wavelength methods demonstrate need for in situ measurement vs. fixed models [238,248].	TRL 5–6; demonstrated in testbeds and EB-PBF platforms; adoption in production LPBF increasing but requires per-machine calibration [234,238,248,249].	Best for obtaining quantitative process temperatures, improving physics-based models, enabling calibrated closed-loop control, as absolute temperature accuracy (±28 K) enables physics-model validation and thermal-based control [234,248,249].
Real-Time Closed-Loop Control	Melt-pool thermal emission feedback to modulate laser power; customized LPBF platforms for on-the-fly control [250].	Lab demonstrations of closed-loop power control based on melt-pool emission on custom platforms [250].	Real-time control loops implemented in testbeds; explicit latency and industrial cycle-time scalability not comprehensively reported [250].	Demonstrations limited to custom/test platforms; industrial scale-up evidence limited in reviewed corpus [250].	TRL 4–5; prototype demonstrations with promising regulator designs; widespread factory integration remains active R&D [250].	Best for dynamic compensation of melt pool instabilities (over melt, balling) in research or pilot production contexts on custom testbeds with specific materials, as closed-loop feedback demonstrated feasibility for real-time process adjustment in controlled laboratory settings [250].

**Table 12:** Sensor technologies and data integration in PBF digital twins.

Technology/Approach	Specifications and Data Characteristics	Validation Level	Computational Performance	Transferability	Industrial Readiness	Best Use Cases and Rationale
Thermography and Pyrometry	Two-wavelength coaxial imaging pyrometry and off-axis thermal imaging for melt-pool temperature mapping; photodiode pyrometry sampling >100 kHz; datasets reach hundreds of thousands of frames and hundreds of GB [263,264,265].	Correlated to μCT/XCT and operando X-ray radiography for pore/keyhole validation and surface topography prediction [263,264,265].	High sampling (>100 kHz) enables sub-ms feature windows for ML; data volumes reach hundreds of GB per multi-build campaign [264,265,266].	Demonstrated across lab printers and multiple builds; domain shift issues require domain alignment/augmentation [256,266].	TRL 6–7; mature for monitoring; integration with data pipelines shown but FAIR standards incomplete [266,267].	Best for temperature control, melt-pool energy tracking, surface topography inference, and as primary features for defect classification models. Why: High temporal resolution (>100 kHz) enables precise thermal event capture; direct correlation to temperature physics supports model validation [263,264,265].
Acoustic Emission Monitoring	Structure-borne and airborne AE capture rapid mechanical/pressure transients from keyhole collapse and spatter; signals resolved at sub-ms time windows; contribute strongly to pore prediction [265,268,269].	Validated with operando synchrotron X-ray for temporal registration of pore events and XCT comparison for predicted porosity [265,268].	Low data volume relative to imaging; enables ML detection within 0.5–4 ms windows [265,268].	Shown to transfer across experiments but sensitivity to machine acoustic environment and sensor placement reported [266,267].	TRL 5–6; high promise for real-time detection; fewer commercial turnkey AE solutions for PBF than optical tools [267].	Best for fast event detection (keyhole pore formation, spatter), early warning signals for closed-loop corrections. Why: Low data overhead with high temporal sensitivity to transient events; strong correlation to pore formation validated by operando X-ray [265,268,269].
Melt Pool Imaging (Optical, Coaxial, Off-Axis)	High-speed visible/SWIR cameras and coaxial sensors capture morphology, intensity, area; dynamic ROI cameras operate up to 20 kHz [263,270,271].	Co-registered to XCT/μCT and operando X-ray; spatial registration methods map melt pool signatures to part coordinates for topography and defect correlation [263,264,265].	Highest data volumes (10^5^–10^6^ frames per build); GPU pipelines required for real-time processing; continuous capture demands ROI or compression [266,270,271].	Imaging models show reduced performance under different instruments; domain adaptation improves cross-setting accuracy [256,266].	TRL 6–7; widely used in research; scaling to full production requires high-throughput pipelines and ROI strategies [266,271].	Best for morphology-based defect detection, surface topography prediction, training labels for other sensors. Why: Spatial resolution enables precise defect localization; registered imaging provides ground truth for ML training [263,264,265].
Multi-Sensor Fusion Frameworks	Common fusions: thermal + optical + acoustic + photodiode; fusion at data/feature/decision levels using CNNs, LSTMs, ensemble classifiers [264,265,268,269].	Validated against operando X-ray and XCT; multi-modal ML achieves pore F1 up to 0.95, recall 1.0, classification accuracies ≈ 98% [264,265,268].	Fusion improves predictive power but raises synchronization and computational load; ML inference over 0.5–4 ms windows [265,268].	Fusion improves robustness but requires per-machine calibration; domain adaptation pipelines increase reusability (+31% detection without labels) [256,266].	TRL 5–6; research prototypes show closed-loop correction in L-DED and DT architectures; production adoption partial due to integration complexity [256,267,269].	Best for comprehensive defect detection and localization where multiple physical signatures are available. Why: Complementary sensors capture different physics; fusion achieves highest reported defect detection metrics (F1 = 0.95, recall = 1.0) [264,265,268].
Machine Learning for Multi-Sensor Processing	CNN + LSTM hybrids and feature-level fusion; training datasets range from thousands to millions of frames and hundreds of GB; reported 61% UTS prediction error reduction using fused in situ data [256,268,272].	Ground truth: XCT/μCT/metallography/tensile testing; reported classification accuracies ≈ 98% (regime), F1 up to 0.95, defect size classification 98.8% (large pores) [264,265,268,272].	Inference times suitable for ms-scale decision windows; full-build scaling needs GPU acceleration and optimized pipelines [266,268,271].	Models trained on one setting degrade on others; domain adaptation and augmentation recover performance [256,266,272].	TRL 5–6; ML essential for DT updates; publicly shared datasets and HDF5 pipelines improve reproducibility but broader FAIR adoption limited [266,267].	Best for automated defect classification, local mechanical property prediction, inputs to closed-loop controllers. Why: ML extracts complex patterns from multi-modal data; validated 61% error reduction in tensile property prediction [268,272].
Spatial–Temporal Data Registration	Methods combine galvanometer coordinates, laser ON/OFF, camera alignment, ML-based image registration to map sensor records to part coordinates; two-camera (coaxial + off-axis) registration recovers spatial melt pool maps [263,264,265].	Registration validated by predicting layer surface topography and correlating melt pool signatures with XCT porosity maps [263,264,265].	Registration incurs moderate compute but essential for co-registered datasets; synchronization precision down to 50 µs with synchrotron timing [263,265].	Necessary for multi-sensor generalization; registration enables cross-sensor mapping across geometries when scanner coordinates available [256,264,266].	TRL 4–5; critical enabling step for DTs and in situ qualification; implementations exist in research toolchains [263,266].	Best for spatially resolved defect localization, part-level mapping for mechanical property inference. Why: Registration accuracy directly impacts ML model reliability; enables precise correlation between in situ signals and ex situ validation [263,264,265].
FAIR Principles and Data Management	Public release examples: 230 GB HDF5 dataset linking in situ sensor footprints to tensile tests; pipelines process 700 k+ frames for feature extraction [266,272].	Ground truth co-registration and labeling practices exist but no universal metadata/schema standard widely adopted [266,267].	FAIR pipelines reduce retraining time and enable transfer but add overhead for metadata capture and storage [266].	Domain adaptation and open datasets improve reuse and DT reusability across institutions [256,266,272].	TRL 3–5; partial adoption: exemplar datasets released but community standards and wide FAIR compliance remain limited [266,267].	Best for dataset sharing for ML model training, benchmarking DT models, regulatory qualification. Why: Standardized data formats enable reproducibility and cross-institution model validation; 230 GB public dataset demonstrates feasibility [266,272].
Real-Time Processing and Closed-Loop Control	Real-time ML inference on 0.5–4 ms windows; system demonstrations of automated laser power control or defect correction in lab platforms [268,273].	Closed-loop demonstrations in custom platforms and L-DED for defect correction; real-time decision support in digital twins [269,273].	Achieved with GPU-accelerated pipelines and ROI imaging; full-build closed-loop across large parts challenging due to data throughput [266,271,273].	Lab demonstrations transferable to production with engineering investment; standardization and safety qualification outstanding [267,273].	TRL 4–5; early stage for full industrial deployment; specific closed-loop functions (power modulation, local repair) demonstrated [273].	Best for on-the-fly power modulation, defect mitigation actions, probabilistic porosity control in DTs. Why: Demonstrated feasibility of ms-scale closed-loop control; probabilistic DT framework shown for online Bayesian calibration [273].

**Table 13:** AI integration in PBF digital twins.

AI Method/Framework	Application Domain	Physics Integration Level	Sensor Modalities	Validation Method	Computational Efficiency	Transferability	Key Limitations	References
Convolutional Neural Networks (CNNs) and Deep Learning for Defect Detection	Defect detection and classification (porosity, lack of fusion, surface defects, melt pool anomalies); quality assessment; in situ process monitoring; multi-label anomaly detection	Purely data-driven deep learning; transfer learning and self-supervised learning implementations reduce labeling requirements	Optical imagery (powder bed images, melt pool images); multi-modal fusion (visible and infrared cameras); layer-wise optical tomography	Ex situ and in situ validation; 82–99.79% accuracy in defect detection and classification; validated against XCT ground truth data	High: real-time processing with practical latency (under 18 s per layer); enables real-time multi-label anomaly detection and intra-layer closed-loop control	High: transfer learning enables quality monitoring across materials with >92% accuracy; self-supervised learning handles imbalanced datasets without extensive labeling	Requires extensive labeled datasets for supervised methods; limited interpretability in complex architectures; limited to single or specific sensor modalities in some implementations; data scarcity for rare defect types	[6,272,287,289,295,296,297,298,299]
Physics-Informed Neural Networks (PINNs) and Deep Neural Operators for Thermal Prediction	Temperature prediction; full-field thermal modeling; melt pool geometry prediction; parameter identification; high-fidelity melt pool state prediction; closed-loop feedback control; scalable temperature distribution	Hybrid physics-informed deep learning: custom loss functions enforcing physical behavior (PDEs embedded); Fourier neural operators combine physics-based simulations with data-driven learning; graph neural networks (GNNs) learn physics from FEA simulations	Infrared camera data; thermal imaging; physics-informed variables extracted from simulations; GNNs trained on FEA simulation data	Ex situ validation with less than 7% deviation; R^2^ > 0.98 for melt pool geometry; closed-loop feedback control with online calibration; GNN validation with 3.77% MAPE	High: computational time significantly reduced vs. pure FEM/CFD (up to 3900× speed-up); efficient with limited and sparse data; architecture-driven approaches effective with minimal data	High: transferable to different builds without retraining; demonstrated on NIST benchmark parts; geometry-agnostic capabilities; GNNs are multi-laser capable and transferable across geometries	Requires partial experimental data; accuracy depends on physics model fidelity; validation on limited geometries and materials; computational demands for high-fidelity simulations; surrogate accuracy depends on training data quality	[5,220,224,227,280,300,301,302]
Ensemble Methods and Hybrid ML-Physics Models for Process Optimization	Melt pool geometry prediction; defect classification (porosity, melt pool stability); density and hardness prediction; process parameter optimization; robust design optimization	Purely data-driven ML (random forest, XGBoost, extra trees) with physics-augmented data generation and CFD-informed training; Bayesian calibration with stochastic modeling and probabilistic frameworks	Trained on process parameters and simulation/experimental data; melt pool monitoring data; infrared thermal imaging with dimensionality reduction	Ex situ validation; 99.79% accuracy in melt pool stability classification; R^2^ up to 0.95 for density prediction; in situ layer-wise parameter adjustment; probabilistic approaches support robust optimization	High: fast inference enabling online monitoring (0.4 ms in some cases); runtime speed-up of 3900× compared to physics models; surrogate models reduce computation, enabling fast updates	High: transferable to different builds without retraining; demonstrated on Ti-6Al-4V, IN 625, SS316L, AlSi10Mg; model tailored for individual parts with model updating	Requires calibration with experimental data; potential for overfitting with complex models; requires high-quality continuous sensor data; computational demands for frequent updating; uncertainty quantification can increase computational burden	[5,256,296,303,304,305,306,307]
Multi-Modal Sensor Fusion with Deep Learning	Flaw detection; defect identification (subsurface porosity, cracking, keyhole pores); quality assessment; multi-material composition monitoring; comprehensive in situ process monitoring	Hybrid ML-assisted and physics-based sensor fusion; deep learning with multi-modal integration; contrastive loss functions and attention mechanisms	Multi-modal: optical, acoustic emission, thermal imaging (visible and infrared cameras), spectral data, X-ray radiography with spatial–temporal registration	In situ validation; 95–98.5% accuracy in flaw/defect detection; 0.95 F1-score for keyhole pore prediction; validated against XCT ground truth; high temporal resolution monitoring	Medium to High: feature-level fusion balances accuracy and computational cost; high-speed systems enable intra-layer control; computational overhead from sensor synchronization	Moderate: contrastive learning improves multi-material composition monitoring; limited cross-material validation in most studies	High complexity in sensor synchronization and data heterogeneity; challenges in data volume, standardization, and registration errors; limited validation on complex geometries; multi-modal datasets scarce; limited interpretability	[243,268,270,287,295,308,309]
Reinforcement Learning and Model Predictive Control for Adaptive Digital Twins	Laser scan path optimization; thermal uniformity; residual stress reduction; geometry-agnostic melt pool control; real-time parameter adjustment; multi-step temperature tracking; adaptive parameter control; block quality prediction	Hybrid: reinforcement learning with reduced-order simulation and physics-based models; ML surrogate models with MPC frameworks; hybrid RNN/LSTM + reinforcement learning; ML and Bayesian optimization integrated	Real-time sensor feedback for control; thermal sensors for layer-wise control; time-series deep neural networks for MPC; real-time sensor data for temperature prediction	In situ validation; demonstrated error reduction experimentally; MPC achieves precise melt pool temperature tracking and outperforms PID control; real-time parameter tuning via RL	High: framework supports dynamic process optimization; multi-step predictive models facilitate timely regulation; real-time temperature prediction and process optimization	Moderate: geometry-agnostic controller demonstrated; validated on Ti-6Al-4V and AISI 316L; some transferability limitations to different process types (e.g., DED to LPBF); limited cross-material validation	Requires extensive training data for RL; validation on specific processes may limit transferability; physics model accuracy critical for MPC; computational demands for frequent updating; model complexity requires extensive training data; limited interpretability	[7,291,292,310,311,312,313]

**Table 14:** Digital twin implementation in PBF.

Digital Twin Type/Architecture	Core Functions	Model Composition	Sensor and Data Integration Strategy	Validation Approach	Computational Efficiency/Scalability	Industrial Readiness	Key Limitations	References
Hybrid Physics–ML Digital Twins with Adaptive Control and Bayesian Updating	Porosity prediction and control; process parameter optimization; layer-wise model updating; real-time parameter tuning; thermal history control; closed-loop feedback; melt pool consistency and geometric accuracy	Hybrid: physics-based models with Bayesian calibration and probabilistic frameworks; ML surrogates integrated with MPC and reinforcement learning; data-driven adaptive control with G-code manipulation	Infrared thermal imaging with dimensionality reduction; real-time sensor feedback (photodiode, melt pool size sensors, thermal sensors); layer-wise control inputs	Ex situ and in situ validation; layer-wise parameter adjustment; demonstrated process control with reduced defects and improved accuracy; MPC achieves precise temperature tracking	High: surrogate models enable fast updates (runtime speed-up of 3900×); real-time processing with practical latency (under 18 s per layer); efficient framework balancing physics and ML	High: layer-wise laser power adjustment, real-time parameter tuning, G-code manipulation, and closed-loop feedback control demonstrated; supports dynamic process optimization	Requires high-quality continuous sensor data; computational demands for frequent updating and MPC; physics model accuracy critical; limited to single sensor modalities in some implementations; validation on specific processes may limit transferability	[7,256,314,315,316,317,318,319]
Physics-Informed Neural Network (PINN) and Deep Neural Operator Digital Twins	Temperature prediction; full-field thermal modeling; melt pool geometry prediction; parameter identification with reduced data requirements; real-time anomaly prognosis and diagnosis; accelerated process parameter selection	Hybrid physics-informed deep learning: custom loss functions enforcing physical behavior (PDE embedded); physics-based data augmentation; ontology-driven interpretable learning; Fourier neural operators; multi-scale–multiphysics models with ML surrogates	Infrared camera data and thermal imaging; physics-informed variables extracted from simulations; emphasis on interpretable learning; no extensive sensor fusion focus	Ex situ validation with less than 7% deviation; R^2^ > 0.98 for melt pool geometry; in situ validation for real-time anomaly prognosis; modular validation frameworks	High: computational time significantly reduced vs. pure FEM/CFD (up to 3900× speed-up); efficient with limited and sparse data; surrogates enable rapid decision-making across scales	Moderate to High: enables faster simulation and potential for real-time process control; real-time monitoring with interpretable physics integration; surrogates enable rapid decision-making for digital twin applications	Requires partial experimental data; accuracy depends on physics model fidelity; synthetic data may not capture all real-world variability; validation of limited geometries; ontology development requires domain expertise; surrogate accuracy depends on training data quality	[205,220,227,320,321,322,323,324]
Multi-Modal Sensor Fusion and Hybrid CFD/FEM-ML Digital Twins	Flaw and defect detection; quality assessment; comprehensive process monitoring; melt pool width prediction; microstructure evolution; process–structure–property correlation; residual stress prediction	Hybrid: ML-assisted and physics-based sensor fusion with deep learning; combines physics-based simulations (CFD/FEM) with data-driven ML components; ROM with ML integration; combines simulated melt pool images with thermal images	Multi-modal: optical imagery, acoustic emission, spectral data, thermal imaging with spatial–temporal registration; combines simulation (CFD/FEM) and experimental data for non-fusion approaches	In situ monitoring validation with 97–98.5% accuracy in defect detection; ex situ and modular validation; accurate prediction of meltpool depth and dendritic spacing; validated against XCT ground truth	Medium to High: computational cost balanced by sensor fusion and ML; physical supervision network reduces CFD cost; hybrid models faster than pure CFD/FEM with 3900× speed-up for surrogates	Moderate to High: validated for in situ monitoring with real-time capability; enables rapid microstructure prediction; model transferable to different builds without retraining in some implementations	High complexity in sensor synchronization and data heterogeneity; computational overhead; not real-time control for CFD/FEM-ML variants; limited validation on complex geometries; surrogate accuracy depends on training data and FEM model fidelity	[5,203,204,231,242,287,325,326,327]
Comprehensive Industrial Digital Twin Frameworks with Knowledge Transfer and Multi-scale Integration	Comprehensive process monitoring, control, optimization, and cyber–physical system integration; knowledge transfer and domain adaptation for reusability; process chain modeling; predictive maintenance; rapid decision-making	Modular hybrid frameworks integrating physics-based and data-driven models; multi-scale–multiphysics models with ML surrogates; domain adaptation techniques for cross-machine/sensor transferability; methodological frameworks for hybrid model construction	Sensor integration emphasized for real-time feedback; multi-modal data in comprehensive frameworks; sensor data domain alignment across different machines and sensor configurations	Modular validation; framework designed for practical industrial deployment; improved anomaly detection accuracy by 31% via domain adaptation; review frameworks discuss implementation challenges and trustworthiness	Framework designed for practical industrial deployment with modular architecture enabling scalability and adaptability; surrogates enable rapid decision-making across scales; enhanced reusability via domain adaptation	High: real-time process monitoring, control components, data analysis, and predictive maintenance; surrogates enable rapid decision-making; enhanced deployment efficiency via domain adaptation; supports scalability and industrial deployment	Limited details on sensor modalities and specific validation in some frameworks; industrial validation needed; integration complexity requires comprehensive infrastructure development; domain shift challenges; methodological frameworks requiring implementation and validation; standardization challenges	[6,293,328,329,330,331,332]

**Table 15:** Overview of topology optimization methods.

Method Category	Physics and Validation	Geometry Complexity	Computational Cost	Transferability	Real-Time Readiness	Limitations	References
SIMP-Based Methods with Overhang Constraints	Geometric overhang angle constraints; penalty formulations; validated on 2D/simplified 3D examples	2D and low-resolution 3D; limited to simplified geometries	Computationally inexpensive; simple penalty parameters	Not reported	Not suitable for real-time control	Trade-offs with structural performance; stress singularities; convergence issues; lacks of thermal/mechanical coupling	[333,334,335,336]
Inherent Strain Method with Residual Stress Modeling	Residual stress and distortion via the inherent strain method; ex situ validation in selected studies; simplified thermal–mechanical coupling	2D and moderate 3D complexity; support structure and topology co-optimization	Fast simulation; reduced adjoint sensitivity cost; parallel computing frameworks accelerate optimization	Limited experimental validation across machines/materials	Not suitable for real-time control due to its iterative nature	Relies on simplified surrogate models; limited accuracy for complex transient effects; high computational cost for high-fidelity coupling	[337,338,339,340,341]
Layer-by-Layer Thermal Process Models	Local layer-wise thermal models; identifies heat concentration zones; thermal overheating constraints; limited in situ validation	High-resolution 2D and 3D; voxel-level simulations; complex geometries supported	High efficiency via parallelization; custom solvers enable fast layer-wise simulation	Not Reported	Potential for near-real-time feedback with further development	Local/layer assumptions may limit accuracy for transient global effects; experimental validation sparse	[342,343,344,345]
Coupled Fictitious Physics and Multi-Constraint Models	Couples geometric constraints (overhang, cavity) with fictitious physics; improved convergence; limited experimental validation	Addresses complex geometric constraints; 2D and 3D examples	Improved convergence; computationally manageable	Not reported	Not suitable for real-time control	User-defined parameters reduce generality; simultaneous multi-constraint control remains challenging	[346,347,348]
Concurrent Topology, Support, and Build Orientation Optimization	Integrates thermal deformation, residual stress, and geometric constraints; surrogate models and homogenization; ex situ validation in few studies	2D and simplified 3D cases; limited applicability to complex industrial parts	High computational cost; surrogate models improve tractability; parallel computing used	Not reported	Not suitable for real-time control	Scalability limited; interaction between support and orientation not fully captured; high runtime	[341,349,350,351,352,353]
Machine Learning-Integrated Methods	ML for turbulent flow modeling and multi-scale lattice optimization; derivative-aware algorithms; limited validation	Applied to niche problems (heat exchangers, lattice scaffolds); not comprehensive	Accelerates specific subproblems; reduces computational burden in targeted applications	Problem-specific; lacks general frameworks and cross-material/machine validation	Early stage; not ready for real-time control	Nascent stage; data requirements; model generalization challenges; coupling with physics-based simulations underdeveloped	[354,355,356]
Multi-Material and Microstructure-Aware Methods	Incorporates material heterogeneity, porosity, graded properties; iterative property updates; limited experimental validation	Simplified material models; small-scale examples	Not reported	Not reported	Not suitable for real-time control	Emerging; complexity of capturing microstructural evolution; sparse experimental validation and process simulation integration	[357,358,359,360,361]

**Table 16:** Process parameter optimization by ML algorithm type.

Process Parameters	Bayesian Optimization	Deep Neural Networks	Traditional ML	Most Effective Algorithm
Laser Power	- Surface quality optimization [362] - Hyperparameter tuning [363]	- Melt pool prediction [230] - Property prediction [364]	- Density optimization [365] - Multi-objective optimization [366]	Bayesian optimization—superior for continuous optimization
Scan Speed	- Process parameter effects [362] - Bead geometry prediction [363]	- Thermal field modeling [141] - Melt pool dynamics [367]	- Relative density prediction [368] - Surface roughness control [369]	Deep neural networks—better for complex relationships
Layer Thickness	- Multi-parameter optimization [362]	- Build quality prediction [370] - Defect detection [371]	- Mechanical properties [372] - Energy consumption [366]	Traditional ML—adequate for linear relationships
Hatch Spacing	- Integrated optimization [362]	- Track morphology [373] - Process monitoring [308]	- Density control [306] - Quality optimization [374]	Bayesian optimization—effective for spacing optimization
Energy Density	- Comprehensive optimization [362]	- Material property prediction [364]	- High-density achievement [365] - Process window determination [375]	Traditional ML—good for energy-based metrics
Multi-Parameter Sets	- BOAT framework [362] - Simultaneous optimization	- Hybrid approaches [370] - Multi-modal fusion [308]	- Ensemble methods [376] - Multi-objective optimization [366]	Bayesian optimization—best for complex multi-parameter spaces

**Table 17:** Build quality metrics and ML outcomes.

Quality Metric	ML Approaches Used	Achieved Improvements	Performance Metrics	Key Findings
Surface Roughness	- Bayesian optimization with transfer learning [362] - Machine learning prediction models [369] - Multi-objective optimization [366]	- Optimized surface quality prediction - Reduced trial-and-error approaches - Real-time quality control	- R^2^ = 98.78% for surface roughness prediction [362] - Significant parameter contribution analysis [369]	Laser power most significant (48.3% contribution) for surface quality [377]
Porosity/Density	- Material-agnostic XGBoost [365] - Gaussian process regression [378,368] - Multiple ML algorithms [306]	- >98% relative density achievement [365] - >99.5% density in EBM [378] - 99.97% maximum density [379]	- Cross-material validation successful [365] - R^2^ = 0.95 (ANN), 0.923 (SVM) [306] - Mean error: 3% for porosity prediction [364]	Material-agnostic approaches show high transferability across alloy systems
Mechanical Properties	- Hybrid neural networks [370] - Deep learning prediction [364] - Evolutionary optimization [366]	- Enhanced tensile property prediction - Improved strength-to-weight ratios - Optimized mechanical performance	- Good accuracy with small training data [370] - Mean error: 0.2% for hardness [364] - 85% correlation coefficient [366]	Microstructure inclusion significantly improves prediction accuracy
Dimensional Accuracy	- Process parameter optimization [372] - Multi-objective approaches [374] - Predictive modeling frameworks [380]	- Reduced dimensional deviations - Improved geometric fidelity - Enhanced manufacturability	- 10% error in predictive models [374] - High accuracy in manufacturability prediction [380]	Systematic parameter optimization reduces dimensional errors significantly
Defect Detection	- Convolutional neural networks [371] - Unsupervised learning [381] - Multi-signal fusion [308]	- 98.63% defect detection accuracy [371] - Real-time monitoring capability - Automated quality assurance	- Minimum defect size: 0.54 mm [371] - 74–99% accuracy for different defect sizes [308]	Feature-level fusion outperforms individual signal-based models
Process Stability	- Real-time monitoring [382] - Acoustic emission analysis [382] - Domain adaptation [383]	- Enhanced process reliability - Reduced build failures - Improved consistency	- 74.39% detection accuracy with LSTM [382] - Successful domain transfer [383]	Multi-modal sensing improves process stability monitoring

Note: ML methods listed here are detailed in Section 5.3. This table focuses on quality metric outcomes and performance indicators.

**Table 18:** Major limitations and open challenges in PBF modeling approaches.

Area of Limitation	Limitation	Papers
Computational Cost and Efficiency	Many thermal and mechanical models for PBF are computationally intensive, limiting their applicability for real-time control or large-scale simulations. This constraint reduces external validity for industrial-scale applications and hinders iterative process optimization.	[2,8,10,314,410,411,412,413,414]
Limited Multi-scale and Multiphysics Integration	Existing models often focus on isolated scales or physics, lacking comprehensive coupling of thermal, mechanical, and microstructural phenomena. This methodological constraint weakens the predictive accuracy of process–structure–property relationships.	[8,9,103,105,415]
Insufficient Experimental Validation	Several modeling approaches rely heavily on simulations with limited experimental calibration or validation, undermining the reliability and generalizability of those findings across different materials and process conditions.	[9,111,122,412,416]
Simplifying Assumptions in Thermal Modeling	Many thermal models assume linearity, flat molten surfaces, or neglect temperature-dependent material properties, which restricts their quantitative accuracy and external validity in capturing real PBF thermal dynamics.	[194,417]
Narrow Focus on Specific Materials or Geometries	Research often concentrates on particular alloys (e.g., Ti-6Al-4V, IN718) or simple geometries, limiting the applicability of models to diverse materials and complex part designs, thus affecting external validity.	[412,417,418,419,420]
Data Limitations for Machine Learning Models	Data-driven and physics-informed neural network models depend on the availability and quality of training data, which is often scarce or expensive to generate, constraining model generalizability and robustness.	[141,301,410,413,414]
Process Parameter Variability and Control Challenges	Variability in process parameters and lack of adaptive control strategies in models limit their ability to predict and mitigate defects dynamically, reducing practical applicability for quality assurance.	[314,411,421,422]
Limited Comparative Studies Across AM Techniques	Few studies comprehensively compare PBF modeling approaches with those of other AM techniques, such as SLA and FDM, restricting broader contextual understanding and cross-technology insights.	[103,423,424,425]

## Data Availability

No new data were created or analyzed in this study. Data sharing is not applicable to this article.

## Abbreviations

The following abbreviations are used in this manuscript:

3D	Three-Dimensional
AI	Artificial Intelligence
AM	Additive Manufacturing
ANN	Artificial Neural Network
API	Application Programming Interface
AR	Augmented Reality
ASTM	American Society for Testing and Materials
CA	Cellular Automaton
CAD	Computer-Aided Design
CET	Columnar-to-Equiaxed Transition
CFD	Computational Fluid Dynamics
CNN	Convolutional Neural Network
CPFE	Crystal Plastivity Finite Element
CT	Computed Tomography
CW	Continuous Wave
DEM	Discrete Element Method
DfAM	Design for Additive Manufacturing
DMLS	Direct Metal Laser Sintering
DOE	Design of Experiments
DT	Digital Twin
EBSD	Electron Backscatter Diffraction
EBM	Electron Beam Melting
ERP	Enterprise Resource Planning
FAIR	Findable, Accessible, Interoperable, Reusable
FCC	Face-Centered Cubic
FDM	Fused Deposition Modeling
FEM	Finite Element Method
FGM	Functionally Graded Material
FVM	Finite Volume Method
GAN	Generative Adversarial Network
GNN	Graph Neural Network
GPU	Graphics Processing Unit
HCP	Hexagonal Close-Packed
HEA	High-Entropy Alloy
ICME	Integrated Computational Materials Engineering
IoT	Internet of Things
IPF	Inverse Pole Figure
IR	Infrared
ISO	International Organization for Standardization
LBM	Lattice Boltzmann Method
LPBF	Laser–Powder Bed Fusion
LSTM	Long Short-Term Memory
LWIR	Long-Wave Infrared
MAPE	Mean Absolute Percentage Error
MES	Manufacturing Execution System
MJF	Multi Jet Fusion
ML	Machine Learning
MPC	Model Predictive Control
MQTT	Message Queuing Telemetry Transport
NDE	Non-Destructive Evaluation
NIR	Near-Infrared
OPC-UA	Open Platform Communications - Unified Architecture
PBF	Powder Bed Fusion
PDE	Partial Differential Equation
PID	Proportional–Integral–Derivative
PINN	Physics-Informed Neural Network
PLM	Product Lifecycle Management
POD	Proper Orthogonal Decomposition/Probability of Detection
PRISMA	Preferred Reporting Items for Systematic Reviews and Meta-Analyses
PSP	Process–Structure–Property
RBF	Radial Basis Function
RL	Reinforcement Learning
RNN	Recurrent Neural Network
RSM	Response Surface Methodology
SEM	Scanning Electron Microscopy
SLA	Stereolithography
SLM	Selective Laser Melting
SLS	Selective Laser Sintering
SPH	Smoothed Particle Hydrodynamics
SVM	Support Vector Machine
SWIR	Short-Wave Infrared
TES	Temperature–Emissivity Separation
TO	Topology Optimization
TRL	Technology Readiness Level
XAI	Explainable Artificial Intelligence
XCT	X-ray Computed Tomography
